# Mechanisms Underlying Curcumin-Induced Neuroprotection in Cerebral Ischemia

**DOI:** 10.3389/fphar.2022.893118

**Published:** 2022-04-26

**Authors:** Feng Fan, Meng Lei

**Affiliations:** ^1^ Department of Interventional Neuroradiology, The First Affiliated Hospital of Zhengzhou University, Zhengzhou, China; ^2^ Department of Neurology, The Third People’s Hospital of Henan Province, Zhengzhou, China

**Keywords:** cerebral ischemia, curcumin, neuroprotection, oxidative stress, inflammation, blood–brain barrier, apoptosis, mitochondrial dysfunction

## Abstract

Ischemic stroke is the leading cause of death and disability worldwide, and restoring the blood flow to ischemic brain tissues is currently the main therapeutic strategy. However, reperfusion after brain ischemia leads to excessive reactive oxygen species production, inflammatory cell recruitment, the release of inflammatory mediators, cell death, mitochondrial dysfunction, endoplasmic reticulum stress, and blood–brain barrier damage; these pathological mechanisms will further aggravate brain tissue injury, ultimately affecting the recovery of neurological functions. It has attracted the attention of researchers to develop drugs with multitarget intervention effects for individuals with cerebral ischemia. A large number of studies have established that curcumin plays a significant neuroprotective role in cerebral ischemia via various mechanisms, including antioxidation, anti-inflammation, anti-apoptosis, protection of the blood–brain barrier, and restoration of mitochondrial function and structure, restoring cerebral circulation, reducing infarct volume, improving brain edema, promoting blood–brain barrier repair, and improving the neurological functions. Therefore, summarizing the results from the latest literature and identifying the potential mechanisms of action of curcumin in cerebral ischemia will serve as a basis and guidance for the clinical applications of curcumin in the future.

## Introduction

Ischemic stroke is the most common type of stroke and is associated with high mortality and morbidity. Early restoration of blood supply to ischemic tissues is currently an effective treatment strategy that improves the energy metabolism, oxygen supply, and neurological outcomes. At present, recombinant tissue plasminogen activator (r-TPA) is used for thrombolytic therapy; however, with the limitation of usage within 4.5 h after the onset of stroke, only 3–5% of stroke patients meet the criteria and use r-TPA in a timely fashion (Wardlaw et al., 2014; Marlier et al., 2015; Moretti et al., 2015; Campbell et al., 2019; Campbell and Khatri, 2020). Therefore, current research focuses on exploring pathological mechanisms and discovering the novel potential therapeutic targets for cerebral ischemia. Cerebral ischemia causes acute brain injury, while reperfusion results in chronic brain injury. In the acute stage of ischemia, cellular homeostasis and microcirculation are impaired, cell energy metabolism is disrupted, and the structure of the blood–brain barrier (BBB) is destroyed. During the reperfusion period, these structures and functions are not restored; many substances and cells that would not otherwise reach the brain, such as inflammatory cells and macromolecules of inflammatory factors, enter the brain through the damaged BBB. This leads to further aggravation of injury following cerebral ischemia (Pan et al., 2007; Jung et al., 2010; Badruddin et al., 2011). In short, the damage caused by cerebral ischemia and reperfusion involves oxidative stress, apoptosis, the inflammatory response, BBB destruction, and energy metabolism disorder, among other pathological mechanisms. Therefore, it is critical to developing drugs that can intervene with multiple targets.

Curcumin is the most important polyphenol active component of turmeric and is slightly soluble in water but soluble in ethanol and acetone. The ratio of compounds in turmeric is about 5% dimethoxylcurcumin, 15% demethoxylcurcumin, and 80% curcumin. It is challenging to dissolve, extract, and absorb curcumin, resulting in low bioavailability and limited clinical applications (Esatbeyoglu et al., 2012; Kotha and Luthria, 2019). In recent years, numerous drug delivery systems using liposomes, nanoparticles, and microemulsion as carriers have been successfully developed, which significantly increased the solubility, stability, and safety of curcumin, and greatly improved its biological activity in treating or preventing diseases, showing great promise for clinical application (Aggarwal and Sung, 2009; Mahmood et al., 2015; Abd El-Hack et al., 2021; Jabczyk et al., 2021; Feltrin et al., 2022).

As a natural medicine, curcumin has a wide range of beneficial pharmacological activities, including antitumor, anti-inflammatory, antioxidation, anti-apoptosis, etc. (Zhou et al., 2011; Mandal et al., 2020; Fu et al., 2021). Numerous studies have revealed the beneficial role of curcumin in cancer, diabetes, metabolic diseases, autoimmune diseases, atherosclerosis, arthritis, pulmonary diseases, etc (Aggarwal and Harikumar, 2009; Jabczyk et al., 2021; Mahjoob and Stochaj, 2021). Recently, researchers discovered that curcumin also has neuroprotective effects on various neurological diseases, including neuropsychiatric disorders, neurodegenerative diseases, traumatic brain injury, spinal cord injury, and epilepsy (Dhir, 2018; Bhat et al., 2019; Yavarpour-Bali et al., 2019; Yuan et al., 2019; Farkhondeh et al., 2020; Nebrisi, 2021; Lamanna-Rama et al., 2022). The involved mechanisms may include the mediation of neurotransmitters and the hypothalamus-pituitary-adrenal cortex axis, the release of neurotrophic factors, and the promotion of nerve regeneration, thereby influencing a variety of signaling cascades, enhancing vitality and differentiation of neurons, and ultimately enhancing neurological functions (Xu et al., 2006; Srivastava et al., 2018; Ramaholimihaso et al., 2020; Yang et al., 2020; Yang et al., 2021). Multiple *in vitro* and *in vivo* experiments have been carried out to investigate the role and mechanism of curcumin in cerebral ischemia and revealed that curcumin participates in the recovery of ischemic injury by inhibiting the oxidation, apoptosis and inflammation, protecting the BBB, and restoring mitochondrial functions (Ovbiagele, 2008; Bavarsad et al., 2019). A summary of recent studies on curcumin treatment for cerebral ischemia will assist in identifying its shortcomings and benefits, thereby guiding future research studies, clinical translational applications, and the exploration of novel therapeutic strategies for ischemic stroke.

### Mechanisms of Curcumin Against Cerebral Ischemia

Recently, numerous studies have demonstrated the neuroprotective effect of curcumin in cerebral ischemia (Bavarsad et al., 2019; Ułamek-Kozioł et al., 2020; Subedi and Gaire, 2021). Curcumin can attenuate neurological dysfunction, and reduce infarct volume and brain edema, thereby improving the outcome of an ischemic stroke. Various mechanisms are involved, including the inhibition of oxidative stress, inflammation, apoptosis, calcium overload, and endoplasmic reticulum stress, as well as the restoration of BBB, and mitochondrial structural functions (Supplementary Table S1). The details are described below.

### Curcumin Reduces Oxidative Stress

Brain tissues have a higher metabolic rate, demand for oxygen and polyunsaturated fatty acids, and lower levels of antioxidant enzymes compared with other organs, making the central nervous system more vulnerable to oxidative damage (Cenini et al., 2019; Torres-Cuevas et al., 2019; Bhatt et al., 2020). Oxidative stress caused by the disruption of homeostasis between oxidative and antioxidant systems are a key mechanism of cerebral ischemic injury (Li et al., 2018; Torres-Cuevas et al., 2019; Yang, 2019). As a vital signaling molecule in the brain, reactive oxygen species (ROS) directly or indirectly mediates several pathological processes after cerebral ischemia (Fraser, 2011; Olmez and Ozyurt, 2012; Orellana-Urzú a et al., 2020). It has been demonstrated that the activity of nitric oxide synthase (NOS), cyclooxygenase (COX), xanthine dehydrogenase/xanthine oxidase, myeloperoxidase, myeloperoxidase (MPO), and other enzymes promoting ROS production increase following stroke, whereas the activity of enzymes that prevent ROS production, such as superoxide dismutase (SOD), catalase, peroxidase, glutathione peroxidase (GSH-Px) decrease, consequently destroying the dynamic balance of ROS, and leading to its accumulation. Excessive ROS can trigger lipid peroxidation, DNA damage, and protein oxidation damage (Sorce et al., 2012; Bazmandegan et al., 2017; Shao et al., 2020; Su et al., 2020; Duan et al., 2021). Therefore, the use of free radical scavengers or other antioxidants is one of the primary therapeutic options for cerebral ischemia (Ahmadinejad et al., 2017; Davis and Pennypacker, 2017; Zhou et al., 2021).

Curcumin, as an antioxidant, accelerates the removal of ROS by activating the antioxidant enzymes and inhibiting the brain tissue damage induced by oxidative stress (Vajragupta et al., 2003; Namgyal et al., 2021). The antioxidative effect of curcumin in cerebral ischemia has been widely explored, and it has been noted that curcumin could partially exert neuroprotection by alleviating oxidative stress-induced injury post-stroke (Rathore et al., 2008; Mukherjee et al., 2019; Zhang et al., 2021). It was previously reported that pretreatment and posttreatment administration of curcumin both improved the antioxidative ability of the injured neurons (Wu et al., 2015), while immediate and delayed (24 h after ischemia) treatments with curcumin both prevented ischemia-induced neuronal damage and oxidative insult, indicating the wide range time window of curcumin treatment in cerebral ischemia (Al-Omar et al., 2006).

Moreover, curcumin can lower the production and accumulation of ROS and oxidation products (MDA, lipid peroxidation, etc.) (Hosseinzadehdehkordi et al., 2015; Seo et al., 2017; Khan et al., 2019). Other formulations of curcumin with polyethylene glycol (PEG)-ylated polylactide-co-glycolide (PLGA) nanoparticles or solid lipid nanoparticles (C-SLNs) are also capable of reducing ROS levels (Mukherjee et al., 2019). Interestingly, a comparative study investigating the antioxidative effect of three curcuminoids (curcumin, demethoxycurcumin, and bisdemethoxycurcumin) using a polymeric N-isopropyl acrylamide nanoparticle formulation determined that curcumin had the most potent antioxidant activity (Ahmad et al., 2013). In addition, curcumin elevates the activity and expression level of antioxidases (NADPH oxidase 2, SOD, CAT, GSH-Px, glutathione reductase, etc.) (Dohare et al., 2008; Kakkar et al., 2013; Wu et al., 2020). Awad *et al.* demonstrated that curcumin synergistically enhanced the inhibitory action of candesartan on brain ischemia through the suppression of oxidative stress, implying the beneficial combined effects and potential therapeutic strategy of curcumin and other drugs on cerebral ischemia in the future (Awad, 2011). Various signaling pathways are involved in curcumin-induced antioxidation. For example, curcumin could alleviate the oxidative damage by regulating the miR-1287-5p/LONP2 axis and miR-7/RelA p65 axis in an OGD/R model (Xu H. et al., 2019; Zhang et al., 2021). Another study described that dienone monocarbonyl curcumin analogs protected the cellular growth by eliminating ROS generation by activating the Nrf2/HO-1 signaling pathway (He et al., 2021). Similarly, curcumin and hexahydrocurcumin enhanced antioxidant defense partially through the Nrf2/HO-1 pathway in a rat stroke model (Wicha et al., 2017). In addition, other signaling pathways such as SP1/Prdx6 (Jia et al., 2017), AMPK/UCP2 (Pu et al., 2013), Golgi reassembly, and stacking protein 65 (GRASP65) (Lin et al., 2016) are also involved in the antioxidant properties of curcumin.

### Curcumin Inhibits Cellular Apoptosis

Apoptosis is an autonomous and programmed process of cell death that is the predominant form of cell death in cerebral ischemia and is closely related to the prognosis of stroke patients (Ferrer and Planas, 2003; Mitsios et al., 2007; Uzdensky, 2019; Gao et al., 2020). Previous research has described that cell necrosis and apoptosis co-exist in the acute stage of cerebral ischemia, while apoptosis is the primary type of delayed cell death post-stroke. Indeed, following the stroke onset, necrosis mainly occurs in the ischemic central region, whereas apoptosis chiefly occurs in the ischemic penumbra (Ueda and Fujita, 2004; Radak et al., 2017). The mechanism of apoptosis induced by cerebral ischemia is intricate and involves not only alterations in the expression of apoptosis-related genes but is also regulated by myriad internal and external factors. The mechanisms that mediate ischemic stroke-induced apoptosis mainly include the mitochondrial and endoplasmic reticulum stress and death receptor pathways (Cao et al., 2001; Zheng et al., 2003; Broughton et al., 2009; Iurlaro and Muñoz-Pinedo, 2016).

The use of anti-apoptotic agents or therapeutic strategies can protect against cell injury after cerebral ischemia (Rami et al., 2008; Luo et al., 2019; Youssef et al., 2021). A large number of studies have reported that various traditional Chinese medicines, including curcumin, can effectively alleviate cellular apoptosis after cerebral ischemia and improve neurologic dysfunction (Dong et al., 2016; Yu et al., 2020; Zhu et al., 2021). Curcumin can upregulate the expression of anti-apoptotic proteins such as Bcl-2 and downregulate the expression of apoptosis-related proteins such as Bax and caspase-3, thus effectively inhibiting cellular apoptosis and attenuating cerebral ischemia-induced injury (Xie et al., 2018; Xu L. et al., 2019). The specific mechanism of curcumin alleviating apoptosis after cerebral ischemia is well-documented. Curcumin-laden exosomes target ischemic brain tissues and alleviate ROS-mediated mitochondrial apoptosis (He et al., 2020). Additionally, curcumin can alleviate ischemia-induced brain injury and cell apoptosis via repressing CCL3, elevating glucose transporter (GLUT)1 and GLUT3, inactivating the TLR4/MyD88/MAPK/NF-κB and Wnt/JNK1 pathways, and promoting MEK/ERK/CREB, and PI3K/Akt pathway activation (Xia et al., 2018; Xu L. et al., 2019; Wang C. et al., 2020; Wu et al., 2020; Zhou et al., 2020). Xu et al. (2018) showed that a combination of curcumin and vagus nerve stimulation restored behavioral deficits by inhibiting apoptosis after cerebral ischemia, with the involvement of the Akt/ERK2 pathway. Notably, curcumin inhibits cellular damage and apoptosis by diminishing the endoplasmic reticulum stress (ERS) (Cheng et al., 2020; Keshk et al., 2020; Zhou et al., 2022). Chhunchha et al. (2013) reported that curcumin abated hypoxia-induced ERS-mediated cell death in mouse hippocampal cells by enhancing peroxiredoxin 6 (Prdx6) expressions and inhibiting NF-κB activation. Another *in vitro* research using the neuroblastoma cells exposed that curcumin relieved neurotoxicity via regulating the PERK-eIF2α pathway (Yan et al., 2022). Last, curcumin mitigated axonal injury and neuronal cellular apoptosis through the PERK/Nrf2 signaling pathway in a rat diffuse axonal injury model (Huang T. et al., 2018).

However, it is worthwhile noting that curcumin could play an antitumor role by promoting the apoptosis of tumor cells (Notarbartolo et al., 2005; Giordano and Tommonaro, 2019; Walker and Mittal, 2020). Furthermore, exploration of the mechanism of curcumin in diverse diseases and its effect on apoptosis under contrasting conditions will assist in evaluating the safety and effectiveness of curcumin treatment in cerebral ischemia in the future.

### Curcumin Diminishes the Inflammatory Cascade

Neuroinflammation plays a key role in the progression of cerebral ischemia. Following cerebral ischemia, microglia, astrocytes, and neutrophils, as the main effector cells, release a large number of inflammatory cytokines, such as interleukins, chemokines, and tumor necrosis factor (TNF), induce neuronal apoptosis, and contribute to microvascular dysfunction, secondary cerebral hemorrhage, and cerebral edema (Wang et al., 2019b; Shi et al., 2019; Jurcau and Simion, 2021). The activation and infiltration of inflammatory cells, as well as the synthesis and secretion of adhesive molecules and inflammatory mediators, promote the inflammatory cascade (Barrington et al., 2017; Hendriksen et al., 2017; Živančević et al., 2021).

Curcumin has been shown to possess anti-inflammatory properties in various neurological disorders, including acute brain injuries (spinal cord injury (Zhang N. et al., 2017), traumatic brain injury (Sun et al., 2020), stroke (Miao et al., 2016), and subarachnoid hemorrhage (Wakade et al., 2009)), and neurodegenerative diseases (Alzheimer’s disease (Hamaguchi et al., 2010), Parkinson’s disease (Ojha et al., 2012), Huntington’s disease (Ullah et al., 2017), and multiple sclerosis (Mohajeri et al., 2015)). It attenuates the inflammatory response after cerebral ischemia through multiple mechanisms. For instance, curcumin can reduce the induction and release inflammatory cytokines such as IL-6, IL-1β, TNF-α, and COX-2 (Zhang Y. et al., 2017; Wicha et al., 2017). In addition, curcuminoids decrease neutrophil rolling and adhesion to the cerebrovascular endothelium, lower neutrophil numbers, and inhibit neutrophil activation, thereby ameliorating ischemic brain injury (Funk et al., 2013). NF-κB is a regulatory factor with diverse transcriptional effects, which are activated after cerebral ischemia and participates in the transcription of relevant target genes contributing to the inflammatory response. Numerous researchers have demonstrated that the anti-inflammatory effect of curcumin in cerebral ischemia is tightly associated with the modulation of NF-κB (Li et al., 2016, 2017; Li et al., 2021). Ran et al. (2021) observed that curcumin ameliorated white matter injury after ischemic stroke via NF-κB suppression and NLRP3 inflammasome inhibition in a rat stroke model. Triblock copolymer nanomicelles loaded with curcumin also exert an anti-inflammatory effect by inhibiting the NF-κB pathway after cerebral ischemia (Li et al., 2021). Other studies assessing the link between NF-κB and curcumin established that the anti-inflammatory impact of curcumin in cerebral ischemia is mediated by the inhibition of the TLR4/MyD88/MAPK/NF-κB, TLR2/NF-κB, and PPAR γ/NF-κB pathways (Liu et al., 2013; Tu et al., 2014; Wang C. et al., 2020). Likewise, the modulation of the TLR4/p38/MAPK, SIRT1 and JAK2/STAT3 pathways (Li L. et al., 2015; Miao et al., 2016; Huang L. et al., 2018) are involved in curcumin-induced inhibition of inflammation in cerebral ischemia. As a recent hotspot area in stroke, ERS also contributes to inflammation and apoptosis in cerebral ischemia. Zhu et al. (2017) described the inhibitory effect of curcumin on ERS by downregulating the expression of GADD153 and caspase-12 in a rat stroke model. Meanwhile, an *in vitro* study exposed that curcumin attenuated neurotoxicity in the hippocampus by suppressing the ERS-associated TXNIP/NLRP3 inflammasome activation in an AMPK-dependent manner (Li Y. et al., 2015).

Microglia are in a resting state under physiological conditions and play the role of “immune monitoring and defense” in the microenvironment of brain cells. Conversely, they are rapidly activated and polarized in the pathological state (Hu et al., 2015; Ma et al., 2017). After the onset of cerebral ischemia, microglia play a contrasting role in brain injury or neuroprotection through M1 or M2 polarization (Xiong et al., 2016; Zhao et al., 2017; Xue et al., 2021). M1 microglia have cytotoxic effects and cause inflammatory tissue damage, whereas M2 microglia have a neuroprotective effect and promote tissue repair and regeneration. The latter congregate in the ischemic area during cerebral ischemia and release inflammatory factors to enhance the inflammatory response. Interestingly, curcumin has a profound regulatory influence on microglial responses, shifting the microglial phenotype from the pro-inflammatory M1 state toward the anti-inflammatory and tissue-reparative M2 phenotype, and inhibiting microglia-mediated pro-inflammatory responses (Hu et al., 2012). The results from both *in vivo* MCAO and *in vitro* OGD models have corroborated that curcumin reduces inflammation through the inhibition of M1 microglial activation and by weakening the increase in TNF-α and IL-1β (Liu et al., 2017; Wang et al., 2019a).

### Curcumin Has a Protective Effect on the Integrity of the BBB

The BBB is predominantly composed of cerebral microvascular endothelial cells, astrocytes, basal lamina, and pericytes. The primary function of BBB is to prevent the diffusion of macromolecules into the brain parenchyma and maintain the stability of the internal environment of the nervous system (Huber et al., 2001; Obermeier et al., 2013; Langen et al., 2019; Alahmari, 2021). After the occurrence of cerebral ischemia, several mediators cause direct damage to the BBB components, which are exacerbated by apoptosis, oxidative stress, and inflammatory reaction, thus increasing the permeability of the BBB and aggravating brain edema and neurologic injury (Jin et al., 2010; Jiang et al., 2018; Kunze and Marti, 2019). Numerous studies have explored the protective role and mechanism of action of curcumin on BBB after ischemic stroke. Curcumin can protect the integrity of BBB and reduce brain edema by the upregulation of aquaporin 4 and tight junction proteins such as zonula occluden 1 (ZO-1), occludin, and claudin-5, and the downregulation of matrix metalloproteinase 9 (MMP-9), intercellular adhesion molecule-l (ICAM-1), and vascular cell adhesion molecule-l (VCAM-1) (Li et al., 2017; Wang et al., 2019a; Wicha et al., 2020; Wu et al., 2021). Furthermore, curcumin attenuates cerebral capillary endothelial cell damage by inhibiting the expression of inducible nitric oxide synthase (iNOS) and the generation of NO(x) (nitrites/nitrates contents), thereby preventing BBB damage (Jiang et al., 2007). The protection of shear rate can also prevent neutrophil adhesion to the cerebrovascular microcirculation and block early microvascular inflammation (Funk et al., 2013). Mo et al. (2021) found that curcumin exhibited a protective effect against cerebral ischemia by reducing the BBB dysfunction through protein kinase C-θ (PKC-θ) signaling. In addition, it was previously reported that curcumin ameliorates the permeability of the BBB during hypoxia by upregulating the expression of HO-1 in brain microvascular endothelial cells (Wang et al., 2013).

Despite many studies demonstrating the protective effect of curcumin on BBB, there are still unanswered questions such as which curcumin formulations and routes of administration can penetrate the BBB more rapidly. What is the main mechanism through which curcumin prevents BBB injury and how to determine the optimal dose and administration interval with favorable safety and efficacy profiles. Further studies are warranted to develop and identify potential treatment strategies for cerebral ischemia.

### Curcumin Improves Mitochondrial Dysfunction and Calcium Overload

The mitochondrion is the main structure for regulating cellular calcium homeostasis. Cellular calcium overload can lead to ROS generation, mainly released from mitochondria, and induce oxidative stress (Kirkinezos and Moraes, 2001; Brookes et al., 2004; Peng and Jou, 2010). Mitochondrial permeability transition pore (mPTP) is a ROS-dependent protein complex between the mitochondrial inner and outer membrane. Calcium overload and oxidative stress in mitochondria can induce the opening of mPTP through lipid peroxidation and mitochondrial respiratory chain damage, thus reducing the mitochondrial membrane potential and releasing cytochrome C (Armstrong, 2006; Rottenberg and Hoek, 2017). The latter is a small molecule protein located in the inner membrane of mitochondria, which serves as an electron carrier between the mitochondrial respiratory chain complex III and complex IV. Its release activates caspase-9, which in turn activates the executor of apoptosis protein caspase-3, and ultimately leads to neuronal apoptosis (Kadenbach et al., 2004; Choi et al., 2007). Therefore, the destruction of mitochondrial structural integrity and functional homeostasis is a significant pathological change in cerebral ischemia injury. Protecting the mitochondrial structure and function is the focus of neuroprotection after cerebral ischemia.

Curcumin can alleviate cerebral ischemic injury by preserving the mitochondrial function and minimizing mitochondrial injury, elevating mitochondrial membrane potential, mitochondrial complex I activity, mitochondrial cytochrome c levels, and maintaining the mitochondrial membrane integrity (Rathore et al., 2008; Kakkar et al., 2013; Miao et al., 2016; Zhang Y. et al., 2017; Wang et al., 2019c). Moreover, curcumin may exert neuroprotective effects by increasing mitochondrial biogenesis, including nuclear respiratory factor-1, mitochondrial transcription factor A, and mitochondrial number (Wang et al., 2005; Liu et al., 2014). He et al. (2020) uncovered that curcumin-laden exosomes alleviated cerebral ischemia-reperfusion injury by inhibiting the ROS-mediated mitochondrial apoptosis. In another study, Mondal et al. (2019) discovered that tetrahydrocurcumin epigenetically mitigated mitochondrial dysfunctions by regulating the mitochondrial tissue inhibitor of metalloproteinase 2 (TIMP-2) through hypermethylation of the CpG islands of TIMP-2 promoter. Furthermore, curcumin can relieve Ca^2+^ dysregulation (Shukla et al., 2008), which may be associated with the inactivation of the P2X7 receptor (Wang Z. et al., 2020). However, the crosstalk and interactions of mitochondrial dysfunction, oxidative stress, calcium overload, and apoptosis in cerebral ischemia are complex. Further research is necessary to reveal the specific neuroprotective mechanism of curcumin in this complicated pathological process.

### Curcumin Regulates Autophagy

Autophagy is a ubiquitous occurrence in eukaryotic animals in which cells phagocytose their own cellular components into vesicles and subsequently fuse with lysosomes to form autophagolysosomes, which breakdown to maintain the cell metabolism and organelle renewal (Mizushima et al., 2008; Mizushima and Komatsu, 2011). It is instrumental in maintaining cell survival and intracellular homeostasis under stressful conditions such as ischemia and hypoxia; however, immoderate autophagy may promote cell death (Smith et al., 2011; Kubisch et al., 2013; Choi et al., 2018). So far, the researchers have detected more than 30 autophagy-related genes involved in regulating autophagy. Cerebral ischemia is known to activate autophagy. However, the role and mechanism of autophagy in cerebral ischemia remain elusive (Wang et al., 2021). The influence and effect of autophagy may be dependent on the degree of ischemic injury and duration of ischemia (Sun et al., 2018; Wang et al., 2018; Wolf et al., 2019; Hou et al., 2022).

Curcumin can exert a beneficial impact by mediating autophagy, thereby inducing antitumor (Masuelli et al., 2017), anti-fibrotic (Kong et al., 2020), anti-apoptotic (Chen et al., 2021), and neuroprotective effects (Forouzanfar et al., 2020). Many studies have illustrated that curcumin attenuates cerebral ischemic injury with the involvement of autophagy. Curcumin can exert neuroprotective effects by suppressing the overactivated autophagy, with a diminished LC3-II/LC3-I ratio (Tyagi et al., 2012; Huang L. et al., 2018; Zhang et al., 2018). Conversely, other researchers hypothesize that curcumin attenuates cerebral ischemia-reperfusion injury by improving mitophagy, with an elevated LC3-II/LC3-I ratio (Wang and Xu, 2020). The difference between curcumin on autophagy may be correlated with the administration time point and dosage of curcumin, the stage of ischemic injury, and other factors. The dynamic alterations in autophagy regulated by curcumin in cerebral ischemia need to be explored in further research. Interestingly, Hou et al. (2019) identified that inhibition of autophagy caused a decrease in HIF-1α and an attenuation in HIF-1α induced autophagy suppression under OGD/R conditions, indicating the importance of the interaction of autophagy and HIF-1α underlying curcumin-induced neuroprotection in brain ischemia.

## Summary

Turmeric is a traditional Chinese medicine widely used in food and medicine and has been used to treat various diseases for millennia. Akin to many natural products, turmeric has a variety of biological activities with low toxicity. As a critical active component of turmeric, curcumin has been found to play a neuroprotective role in the treatment of cerebral ischemia through various mechanisms, such as antioxidant activity, anti-apoptosis, anti-inflammatory activity, and BBB protection. However, there are unresolved questions. First, the clinical application of curcumin is challenging. At present, most of the studies are experimental by nature, and related clinical trials are limited. Although basic research has achieved favorable results, it should be noted that animals and humans have significant differences in terms of drug applications, such as drug dosage and frequency, administration route, and treatment time points. In addition, it has a strong desire to further illustrate the effectiveness, safety, and stability of curcumin in the body through clinical trials, and choose the optimal treatment strategy. Second, the effect of curcumin combined with other drugs and treatment methods should be explored to determine the potential mechanism of their synergistic effects in promoting the therapeutic effect of curcumin. Furthermore, curcumin has a wide range of therapeutic targets, making it challenging to focus on just one. Therefore, an effective strategy to maximize the efficacy of curcumin is by accelerating the development of drug delivery systems based on nanoparticles and other carriers and to carry out targeted modification in the new forms of curcumin. Last but not least, it is imperative to further deepen our understanding of the biological and pharmacological activities of curcumin. Considering that curcumin is almost insoluble in water and has a short half-life and low bioavailability, further studies are warranted to determine its application in cerebral ischemic therapy.

## References

[B1] Abd El-Hack M. E. El-Saadony M. T. Swelum A. A. Arif M. Abo Ghanima M. M. Shukry M. (2021). Curcumin, the Active Substance of Turmeric: its Effects on Health and Ways to Improve its Bioavailability. J. Sci. Food Agric. 101, 5747–5762. 10.1002/jsfa.11372 34143894

[B2] Aggarwal B. B. Harikumar K. B. (2009). Potential Therapeutic Effects of Curcumin, the Anti-inflammatory Agent, against Neurodegenerative, Cardiovascular, Pulmonary, Metabolic, Autoimmune and Neoplastic Diseases. Int. J. Biochem. Cel Biol 41, 40–59. 10.1016/j.biocel.2008.06.010 PMC2637808 18662800

[B3] Aggarwal B. B. Sung B. (2009). Pharmacological Basis for the Role of Curcumin in Chronic Diseases: an Age-Old Spice with Modern Targets. Trends Pharmacol. Sci. 30, 85–94. 10.1016/j.tips.2008.11.002 19110321

[B4] Ahmad N. Umar S. Ashafaq M. Akhtar M. Iqbal Z. Samim M. (2013). A Comparative Study of PNIPAM Nanoparticles of Curcumin, Demethoxycurcumin, and Bisdemethoxycurcumin and Their Effects on Oxidative Stress Markers in Experimental Stroke. Protoplasma 250, 1327–1338. 10.1007/s00709-013-0516-9 23784381

[B5] Ahmadinejad F. Geir Møller S. Hashemzadeh-Chaleshtori M. Bidkhori G. Jami M. S. (2017). Molecular Mechanisms behind Free Radical Scavengers Function against Oxidative Stress. Antioxidants (Basel) 6, 51. 10.3390/antiox6030051 PMC5618079 28698499

[B6] Al-Omar F. A. Nagi M. N. Abdulgadir M. M. Al Joni K. S. Al-Majed A. A. (2006). Immediate and Delayed Treatments with Curcumin Prevents Forebrain Ischemia-Induced Neuronal Damage and Oxidative Insult in the Rat hippocampus. Neurochem. Res. 31, 611–618. 10.1007/s11064-006-9059-1 16770732

[B7] Alahmari A. (2021). Blood-Brain Barrier Overview: Structural and Functional Correlation. Neural Plast. 2021, 6564585. 10.1155/2021/6564585 34912450 PMC8668349

[B8] Altinay S. Cabalar M. Isler C. Yildirim F. Celik D. S. Zengi O. (2017). Is Chronic Curcumin Supplementation Neuroprotective against Ischemia for Antioxidant Activity, Neurological Deficit, or Neuronal Apoptosis in an Experimental Stroke Model? Turk Neurosurg. 27, 537–545. 10.5137/1019-5149.JTN.17405-16.0 27593816

[B9] Armstrong J. S. (2006). The Role of the Mitochondrial Permeability Transition in Cell Death. Mitochondrion 6, 225–234. 10.1016/j.mito.2006.07.006 16935572

[B10] Awad A. S. (2011). Effect of Combined Treatment with Curcumin and Candesartan on Ischemic Brain Damage in Mice. J. Stroke Cerebrovasc. Dis. 20, 541–548. 10.1016/j.jstrokecerebrovasdis.2010.03.008 20719539

[B11] Badruddin A. Taqi M. A. Abraham M. G. Dani D. Zaidat O. O. (2011). Neurocritical Care of a Reperfused Brain. Curr. Neurol. Neurosci. Rep. 11, 104–110. 10.1007/s11910-010-0156-9 21042886

[B12] Barrington J. Lemarchand E. Allan S. M. (2017). A Brain in Flame; Do Inflammasomes and Pyroptosis Influence Stroke Pathology? Brain Pathol. 27, 205–212. 10.1111/bpa.12476 27997059 PMC8028888

[B13] Bavarsad K. Barreto G. E. Hadjzadeh M. A. Sahebkar A. (2019). Protective Effects of Curcumin against Ischemia-Reperfusion Injury in the Nervous System. Mol. Neurobiol. 56, 1391–1404. 10.1007/s12035-018-1169-7 29948942

[B14] Bazmandegan G. Boroushaki M. T. Shamsizadeh A. Ayoobi F. Hakimizadeh E. Allahtavakoli M. (2017). Brown Propolis Attenuates Cerebral Ischemia-Induced Oxidative Damage via Affecting Antioxidant Enzyme System in Mice. Biomed. Pharmacother. 85, 503–510. 10.1016/j.biopha.2016.11.057 27889229

[B15] Bhat A. Mahalakshmi A. M. Ray B. Tuladhar S. Hediyal T. A. Manthiannem E. (2019). Benefits of Curcumin in Brain Disorders. Biofactors 45, 666–689. 10.1002/biof.1533 31185140

[B16] Bhatt S. Nagappa A. N. Patil C. R. (2020). Role of Oxidative Stress in Depression. Drug Discov. Today 25, 1270–1276. 10.1016/j.drudis.2020.05.001 32404275

[B17] Brookes P. S. Yoon Y. Robotham J. L. Anders M. W. Sheu S. S. (2004). Calcium, ATP, and ROS: a Mitochondrial Love-Hate triangle. Am. J. Physiol. Cel Physiol 287, C817–C833. 10.1152/ajpcell.00139.2004 15355853

[B18] Broughton B. R. Reutens D. C. Sobey C. G. (2009). Apoptotic Mechanisms after Cerebral Ischemia. Stroke 40, e331–9. 10.1161/STROKEAHA.108.531632 19182083

[B19] Campbell B. C. V. De Silva D. A. Macleod M. R. Coutts S. B. Schwamm L. H. Davis S. M. (2019). Ischaemic Stroke. Nat. Rev. Dis. Primers 5, 70. 10.1038/s41572-019-0118-8 31601801

[B20] Campbell B. C. V. Khatri P. (2020). Stroke. The Lancet 396, 129–142. 10.1016/s0140-6736(20)31179-x 32653056

[B21] Cao G. Minami M. Pei W. Yan C. Chen D. O'horo C. (2001). Intracellular Bax Translocation after Transient Cerebral Ischemia: Implications for a Role of the Mitochondrial Apoptotic Signaling Pathway in Ischemic Neuronal Death. J. Cereb. Blood Flow Metab. 21, 321–333. 10.1097/00004647-200104000-00001 11323518

[B22] Cenini G. Lloret A. Cascella R. (2019). Oxidative Stress in Neurodegenerative Diseases: From a Mitochondrial Point of View. Oxid Med. Cel Longev 2019, 2105607. 10.1155/2019/2105607 PMC6532273 31210837

[B23] Chen T. Zhou R. Chen Y. Fu W. Wei X. Ma G. (2021). Curcumin Ameliorates IL-1β-induced Apoptosis by Activating Autophagy and Inhibiting the NF-Κb Signaling Pathway in Rat Primary Articular Chondrocytes. Cell Biol Int 45, 976–988. 10.1002/cbin.11541 33377585

[B24] Cheng T. Zhang Z. Shen H. Jian Z. Li J. Chen Y. (2020). Topically Applicated Curcumin/gelatin-Blended Nanofibrous Mat Inhibits Pancreatic Adenocarcinoma by Increasing ROS Production and Endoplasmic Reticulum Stress Mediated Apoptosis. J. Nanobiotechnology 18, 126. 10.1186/s12951-020-00687-2 32891174 PMC7487882

[B25] Chhunchha B. Fatma N. Kubo E. Rai P. Singh S. P. Singh D. P. (2013). Curcumin Abates Hypoxia-Induced Oxidative Stress Based-ER Stress-Mediated Cell Death in Mouse Hippocampal Cells (HT22) by Controlling Prdx6 and NF-Κb Regulation. Am. J. Physiol. Cel Physiol 304, C636–C655. 10.1152/ajpcell.00345.2012 PMC3625714 23364261

[B26] Choi S. Y. Gonzalvez F. Jenkins G. M. Slomianny C. Chretien D. Arnoult D. (2007). Cardiolipin Deficiency Releases Cytochrome C from the Inner Mitochondrial Membrane and Accelerates Stimuli-Elicited Apoptosis. Cell Death Differ 14, 597–606. 10.1038/sj.cdd.4402020 16888643

[B27] Choi Y. Bowman J. W. Jung J. U. (2018). Autophagy during Viral Infection - a Double-Edged Sword. Nat. Rev. Microbiol. 16, 341–354. 10.1038/s41579-018-0003-6 29556036 PMC6907743

[B28] Davis S. M. Pennypacker K. R. (2017). Targeting Antioxidant Enzyme Expression as a Therapeutic Strategy for Ischemic Stroke. Neurochem. Int. 107, 23–32. 10.1016/j.neuint.2016.12.007 28043837 PMC5461189

[B29] de Alcântara G. F. Simões-Neto E. Da Cruz G. M. Nobre M. E. Neves K. R. De Andrade G. M. (2017). Curcumin Reverses Neurochemical, Histological and Immuno-Histochemical Alterations in the Model of Global Brain Ischemia. J. Tradit Complement. Med. 7, 14–23. 10.1016/j.jtcme.2015.10.001 28053883 PMC5198799

[B30] Dhir A. (2018). Curcumin in Epilepsy Disorders. Phytother Res. 32, 1865–1875. 10.1002/ptr.6125 29917276

[B31] Dohare P. Garg P. Jain V. Nath C. Ray M. (2008). Dose Dependence and Therapeutic Window for the Neuroprotective Effects of Curcumin in Thromboembolic Model of Rat. Behav. Brain Res. 193, 289–297. 10.1016/j.bbr.2008.06.012 18611416

[B32] Dong H. J. Shang C. Z. Peng D. W. Xu J. Xu P. X. Zhan L. (2014). Curcumin Attenuates Ischemia-like Injury Induced IL-1β Elevation in Brain Microvascular Endothelial Cells via Inhibiting MAPK Pathways and Nuclear Factor-Κb Activation. Neurol. Sci. 35, 1387–1392. 10.1007/s10072-014-1718-4 24651933

[B33] Dong Q. Lin X. Shen L. Feng Y. (2016). The Protective Effect of Herbal Polysaccharides on Ischemia-Reperfusion Injury. Int. J. Biol. Macromol 92, 431–440. 10.1016/j.ijbiomac.2016.07.052 27431791

[B34] Duan J. Gao S. Tu S. Lenahan C. Shao A. Sheng J. (2021). Pathophysiology and Therapeutic Potential of NADPH Oxidases in Ischemic Stroke-Induced Oxidative Stress. Oxid Med. Cel Longev 2021, 6631805. 10.1155/2021/6631805 PMC7969100 33777315

[B35] Esatbeyoglu T. Huebbe P. Ernst I. M. Chin D. Wagner A. E. Rimbach G. (2012). Curcumin--from Molecule to Biological Function. Angew. Chem. Int. Ed. Engl. 51, 5308–5332. 10.1002/anie.201107724 22566109

[B36] Farkhondeh T. Samarghandian S. Roshanravan B. Peivasteh-Roudsari L. (2020). Impact of Curcumin on Traumatic Brain Injury and Involved Molecular Signaling Pathways. Recent Pat Food Nutr. Agric. 11, 137–144. 10.2174/2212798410666190617161523 31288732

[B37] Feltrin F. D. S. Agner T. Sayer C. Lona L. M. F. (2022). Curcumin Encapsulation in Functional PLGA Nanoparticles: A Promising Strategy for Cancer Therapies. Adv. Colloid Interf. Sci 300, 102582. 10.1016/j.cis.2021.102582 34953375

[B38] Ferrer I. Planas A. M. (2003). Signaling of Cell Death and Cell Survival Following Focal Cerebral Ischemia: Life and Death Struggle in the Penumbra. J. Neuropathol. Exp. Neurol. 62, 329–339. 10.1093/jnen/62.4.329 12722825

[B39] Forouzanfar F. Read M. I. Barreto G. E. Sahebkar A. (2020). Neuroprotective Effects of Curcumin through Autophagy Modulation. IUBMB Life 72, 652–664. 10.1002/iub.2209 31804772

[B40] Fraser P. A. (2011). The Role of Free Radical Generation in Increasing Cerebrovascular Permeability. Free Radic. Biol. Med. 51, 967–977. 10.1016/j.freeradbiomed.2011.06.003 21712087

[B41] Fu Y. S. Chen T. H. Weng L. Huang L. Lai D. Weng C. F. (2021). Pharmacological Properties and Underlying Mechanisms of Curcumin and Prospects in Medicinal Potential. Biomed. Pharmacother. 141, 111888. 10.1016/j.biopha.2021.111888 34237598

[B42] Funk J. L. Frye J. B. Davis-Gorman G. Spera A. L. Bernas M. J. Witte M. H. (2013). Curcuminoids Limit Neutrophil-Mediated Reperfusion Injury in Experimental Stroke by Targeting the Endothelium. Microcirculation 20, 544–554. 10.1111/micc.12054 23464666 PMC3740077

[B43] Gao S. Fang Y. Tu S. Chen H. Shao A. (2020). Insight into the Divergent Role of TRAIL in Non-neoplastic Neurological Diseases. J. Cel Mol Med 24, 11070–11083. 10.1111/jcmm.15757 PMC7576257 32827246

[B44] Gim S. A. Lee S. R. Shah F. A. Koh P. O. (2015). Curcumin Attenuates the Middle Cerebral Artery Occlusion-Induced Reduction in γ-enolase Expression in an Animal Model. Lab. Anim. Res. 31, 198–203. 10.5625/lar.2015.31.4.198 26755923 PMC4707148

[B45] Giordano A. Tommonaro G. (2019). Curcumin and Cancer. Nutrients 11, 2376. 10.3390/nu11102376 PMC6835707 31590362

[B46] Hamaguchi T. Ono K. Yamada M. (2010). REVIEW: Curcumin and Alzheimer's Disease. CNS Neurosci. Ther. 16, 285–297. 10.1111/j.1755-5949.2010.00147.x 20406252 PMC6493893

[B47] He R. Jiang Y. Shi Y. Liang J. Zhao L. (2020). Curcumin-laden Exosomes Target Ischemic Brain Tissue and Alleviate Cerebral Ischemia-Reperfusion Injury by Inhibiting ROS-Mediated Mitochondrial Apoptosis. Mater. Sci. Eng. C Mater. Biol. Appl. 117, 111314. 10.1016/j.msec.2020.111314 32919674

[B48] He W. Wang J. Jin Q. Zhang J. Liu Y. Jin Z. (2021). Design, green Synthesis, Antioxidant Activity Screening, and Evaluation of Protective Effect on Cerebral Ischemia Reperfusion Injury of Novel Monoenone Monocarbonyl Curcumin Analogs. Bioorg. Chem. 114, 105080. 10.1016/j.bioorg.2021.105080 34225164

[B49] Hendriksen E. Van Bergeijk D. Oosting R. S. Redegeld F. A. (2017). Mast Cells in Neuroinflammation and Brain Disorders. Neurosci. Biobehav Rev. 79, 119–133. 10.1016/j.neubiorev.2017.05.001 28499503

[B50] Hosseinzadehdehkordi M. Adelinik A. Tashakor A. (2015). Dual Effect of Curcumin Targets Reactive Oxygen Species, Adenosine Triphosphate Contents and Intermediate Steps of Mitochondria-Mediated Apoptosis in Lung Cancer Cell Lines. Eur. J. Pharmacol. 769, 203–210. 10.1016/j.ejphar.2015.11.019 26593433

[B51] Hou W. Hao Y. Sun L. Zhao Y. Zheng X. Song L. (2022). The Dual Roles of Autophagy and the GPCRs-Mediating Autophagy Signaling Pathway after Cerebral Ischemic Stroke. Mol. Brain 15, 14. 10.1186/s13041-022-00899-7 35109896 PMC8812204

[B52] Hou Y. Wang J. Feng J. (2019). The Neuroprotective Effects of Curcumin Are Associated with the Regulation of the Reciprocal Function between Autophagy and HIF-1α in Cerebral Ischemia-Reperfusion Injury. Drug Des. Devel Ther. 13, 1135–1144. 10.2147/DDDT.S194182 PMC6461000 31040648

[B53] Hu X. Leak R. K. Shi Y. Suenaga J. Gao Y. Zheng P. (2015). Microglial and Macrophage Polarization—New Prospects for Brain Repair. Nat. Rev. Neurol. 11, 56–64. 10.1038/nrneurol.2014.207 25385337 PMC4395497

[B54] Hu X. Li P. Guo Y. Wang H. Leak R. K. Chen S. (2012). Microglia/macrophage Polarization Dynamics Reveal Novel Mechanism of Injury Expansion after Focal Cerebral Ischemia. Stroke 43, 3063–3070. 10.1161/STROKEAHA.112.659656 22933588

[B55] Huang L. Chen C. Zhang X. Li X. Chen Z. Yang C. (2018). Neuroprotective Effect of Curcumin against Cerebral Ischemia-Reperfusion via Mediating Autophagy and Inflammation. J. Mol. Neurosci. 64, 129–139. 10.1007/s12031-017-1006-x 29243061

[B56] Huang L. Li X. Liu Y. Liang X. Ye H. Yang C. (2021). Curcumin Alleviates Cerebral Ischemia-Reperfusion Injury by Inhibiting NLRP1-dependent Neuronal Pyroptosis. Curr. Neurovasc Res. 18, 189–196. 10.2174/1567202618666210607150140 34109908

[B57] Huang T. Zhao J. Guo D. Pang H. Zhao Y. Song J. (2018). Curcumin Mitigates Axonal Injury and Neuronal Cell Apoptosis through the PERK/Nrf2 Signaling Pathway Following Diffuse Axonal Injury. Neuroreport 29, 661–677. 10.1097/WNR.0000000000001015 29570500 PMC5959262

[B58] Huber J. D. Egleton R. D. Davis T. P. (2001). Molecular Physiology and Pathophysiology of Tight Junctions in the Blood-Brain Barrier. Trends Neurosci. 24, 719–725. 10.1016/s0166-2236(00)02004-x 11718877

[B59] Iurlaro R. Muñoz-Pinedo C. (2016). Cell Death Induced by Endoplasmic Reticulum Stress. Febs j 283, 2640–2652. 10.1111/febs.13598 26587781

[B60] Jabczyk M. Nowak J. Hudzik B. Zubelewicz-Szkodzińska B. (2021). Curcumin in Metabolic Health and Disease. Nutrients 13, 4440. 10.3390/nu13124440 34959992 PMC8706619

[B61] Jia G. Tan B. Ma J. Zhang L. Jin X. Li C. (2017). Prdx6 Upregulation by Curcumin Attenuates Ischemic Oxidative Damage via SP1 in Rats after Stroke. Biomed. Res. Int. 2017, 6597401. 10.1155/2017/6597401 28596967 PMC5449737

[B62] Jiang J. Wang W. Sun Y. J. Hu M. Li F. Zhu D. Y. (2007). Neuroprotective Effect of Curcumin on Focal Cerebral Ischemic Rats by Preventing Blood-Brain Barrier Damage. Eur. J. Pharmacol. 561, 54–62. 10.1016/j.ejphar.2006.12.028 17303117

[B63] Jiang X. Andjelkovic A. V. Zhu L. Yang T. Bennett M. V. L. Chen J. (2018). Blood-brain Barrier Dysfunction and Recovery after Ischemic Stroke. Prog. Neurobiol. 163-164, 144–171. 10.1016/j.pneurobio.2017.10.001 28987927 PMC5886838

[B64] Jin R. Yang G. Li G. (2010). Molecular Insights and Therapeutic Targets for Blood-Brain Barrier Disruption in Ischemic Stroke: Critical Role of Matrix Metalloproteinases and Tissue-type Plasminogen Activator. Neurobiol. Dis. 38, 376–385. 10.1016/j.nbd.2010.03.008 20302940 PMC2862862

[B65] Jung J. E. Kim G. S. Chen H. Maier C. M. Narasimhan P. Song Y. S. (2010). Reperfusion and Neurovascular Dysfunction in Stroke: from Basic Mechanisms to Potential Strategies for Neuroprotection. Mol. Neurobiol. 41, 172–179. 10.1007/s12035-010-8102-z 20157789 PMC2877155

[B66] Jurcau A. Simion A. (2021). Neuroinflammation in Cerebral Ischemia and Ischemia/Reperfusion Injuries: From Pathophysiology to Therapeutic Strategies. Int. J. Mol. Sci. 23, 14. 10.3390/ijms23010014 35008440 PMC8744548

[B67] Kadenbach B. Arnold S. Lee I. Hüttemann M. (2004). The Possible Role of Cytochrome C Oxidase in Stress-Induced Apoptosis and Degenerative Diseases. Biochim. Biophys. Acta 1655, 400–408. 10.1016/j.bbabio.2003.06.005 15100056

[B68] Kakkar V. Muppu S. K. Chopra K. Kaur I. P. (2013). Curcumin Loaded Solid Lipid Nanoparticles: an Efficient Formulation Approach for Cerebral Ischemic Reperfusion Injury in Rats. Eur. J. Pharm. Biopharm. 85, 339–345. 10.1016/j.ejpb.2013.02.005 23454202

[B69] Kalani A. Chaturvedi P. Kamat P. K. Maldonado C. Bauer P. Joshua I. G. (2016). Curcumin-loaded Embryonic Stem Cell Exosomes Restored Neurovascular Unit Following Ischemia-Reperfusion Injury. Int. J. Biochem. Cel Biol 79, 360–369. 10.1016/j.biocel.2016.09.002 PMC5067233 27594413

[B70] Kamali Dolatabadi L. Emamghoreishi M. Namavar M. R. Badeli Sarkala H. (2019). Curcumin Effects on Memory Impairment and Restoration of Irregular Neuronal Distribution in the Hippocampal CA1 Region after Global Cerebral Ischemia in Male Rats. Basic Clin. Neurosci. 10, 527–539. 10.32598/bcn.9.10.365 32284841 PMC7149957

[B71] Keshk W. A. Elseady W. S. Sarhan N. I. Zineldeen D. H. (2020). Curcumin Attenuates Cytoplasmic/endoplasmic Reticulum Stress, Apoptosis and Cholinergic Dysfunction in Diabetic Rat hippocampus. Metab. Brain Dis. 35, 637–647. 10.1007/s11011-020-00551-0 32172517

[B72] Khan H. Ullah H. Nabavi S. M. (2019). Mechanistic Insights of Hepatoprotective Effects of Curcumin: Therapeutic Updates and Future Prospects. Food Chem. Toxicol. 124, 182–191. 10.1016/j.fct.2018.12.002 30529260

[B73] Kirkinezos I. G. Moraes C. T. (2001). Reactive Oxygen Species and Mitochondrial Diseases. Semin. Cel Dev Biol 12, 449–457. 10.1006/scdb.2001.0282 11735379

[B74] Kong D. Zhang Z. Chen L. Huang W. Zhang F. Wang L. (2020). Curcumin Blunts Epithelial-Mesenchymal Transition of Hepatocytes to Alleviate Hepatic Fibrosis through Regulating Oxidative Stress and Autophagy. Redox Biol. 36, 101600. 10.1016/j.redox.2020.101600 32526690 PMC7287144

[B75] Kotha R. R. Luthria D. L. (2019). Curcumin: Biological, Pharmaceutical, Nutraceutical, and Analytical Aspects. Molecules 24, 2930. 10.3390/molecules24162930 PMC6720683 31412624

[B76] Kubisch J. Türei D. Földvári-Nagy L. Dunai Z. A. Zsákai L. Varga M. (2013). Complex Regulation of Autophagy in Cancer - Integrated Approaches to Discover the Networks that Hold a Double-Edged Sword. Semin. Cancer Biol. 23, 252–261. 10.1016/j.semcancer.2013.06.009 23810837

[B77] Kunze R. Marti H. H. (2019). Angioneurins - Key Regulators of Blood-Brain Barrier Integrity during Hypoxic and Ischemic Brain Injury. Prog. Neurobiol. 178, 101611. 10.1016/j.pneurobio.2019.03.004 30970273

[B78] Lamanna-Rama N. Romero-Miguel D. Desco M. Soto-Montenegro M. L. (2022). An Update on the Exploratory Use of Curcumin in Neuropsychiatric Disorders. Antioxidants (Basel) 11, 353. 10.3390/antiox11020353 35204235 PMC8868558

[B79] Langen U. H. Ayloo S. Gu C. (2019). Development and Cell Biology of the Blood-Brain Barrier. Annu. Rev. Cel Dev Biol 35, 591–613. 10.1146/annurev-cellbio-100617-062608 PMC8934576 31299172

[B80] Li F. Xu Y. Li X. Wang X. Yang Z. Li W. (2021). Triblock Copolymer Nanomicelles Loaded with Curcumin Attenuates Inflammation via Inhibiting the NF-Κb Pathway in the Rat Model of Cerebral Ischemia. Int. J. Nanomedicine 16, 3173–3183. 10.2147/IJN.S300379 34007172 PMC8121676

[B81] Li L. Li H. Li M. (2015). Curcumin Protects against Cerebral Ischemia-Reperfusion Injury by Activating JAK2/STAT3 Signaling Pathway in Rats. Int. J. Clin. Exp. Med. 8, 14985–14991. 26628981 PMC4658870

[B82] Li P. Stetler R. A. Leak R. K. Shi Y. Li Y. Yu W. (2018). Oxidative Stress and DNA Damage after Cerebral Ischemia: Potential Therapeutic Targets to Repair the Genome and Improve Stroke Recovery. Neuropharmacology 134, 208–217. 10.1016/j.neuropharm.2017.11.011 29128308 PMC5940593

[B83] Li W. Suwanwela N. C. Patumraj S. (2016). Curcumin by Down-Regulating NF-kB and Elevating Nrf2, Reduces Brain Edema and Neurological Dysfunction after Cerebral I/R. Microvasc. Res. 106, 117–127. 10.1016/j.mvr.2015.12.008 26686249

[B84] Li W. Suwanwela N. C. Patumraj S. (2017). Curcumin Prevents Reperfusion Injury Following Ischemic Stroke in Rats via Inhibition of NF-κB, ICAM-1, MMP-9 and C-aspase-3 E-xpression. Mol. Med. Rep. 16, 4710–4720. 10.3892/mmr.2017.7205 28849007 PMC5647023

[B85] Li Y. Li J. Li S. Li Y. Wang X. Liu B. (2015). Curcumin Attenuates Glutamate Neurotoxicity in the hippocampus by Suppression of ER Stress-Associated TXNIP/NLRP3 Inflammasome Activation in a Manner Dependent on AMPK. Toxicol. Appl. Pharmacol. 286, 53–63. 10.1016/j.taap.2015.03.010 25791922

[B86] Lin B. Yu H. Lin Y. Cai C. Lu H. Zhu X. (2016). Suppression of GRASP65 Phosphorylation by Tetrahydrocurcumin Protects against Cerebral Ischemia/reperfusion Injury via ERK Signaling. Mol. Med. Rep. 14, 4775–4780. 10.3892/mmr.2016.5816 27748926

[B87] Liu L. Zhang W. Wang L. Li Y. Tan B. Lu X. (2014). Curcumin Prevents Cerebral Ischemia Reperfusion Injury via Increase of Mitochondrial Biogenesis. Neurochem. Res. 39, 1322–1331. 10.1007/s11064-014-1315-1 24777807

[B88] Liu S. Cao Y. Qu M. Zhang Z. Feng L. Ye Z. (2016). Curcumin Protects against Stroke and Increases Levels of Notch Intracellular Domain. Neurol. Res. 38, 553–559. 10.1080/01616412.2016.1187804 27320251

[B89] Liu Z. Ran Y. Huang S. Wen S. Zhang W. Liu X. (2017). Curcumin Protects against Ischemic Stroke by Titrating Microglia/Macrophage Polarization. Front. Aging Neurosci. 9, 233. 10.3389/fnagi.2017.00233 28785217 PMC5519528

[B90] Liu Z. J. Liu W. Liu L. Xiao C. Wang Y. Jiao J. S. (2013). Curcumin Protects Neuron against Cerebral Ischemia-Induced Inflammation through Improving PPAR-Gamma Function. Evid. Based Complement. Alternat Med. 2013, 470975. 10.1155/2013/470975 23762140 PMC3670515

[B91] Lu Z. Liu Y. Shi Y. Shi X. Wang X. Xu C. (2018). Curcumin Protects Cortical Neurons against Oxygen and Glucose Deprivation/reoxygenation Injury through Flotillin-1 and Extracellular Signal-Regulated Kinase1/2 Pathway. Biochem. Biophys. Res. Commun. 496, 515–522. 10.1016/j.bbrc.2018.01.089 29353037

[B92] Luo Y. Tang H. Li H. Zhao R. Huang Q. Liu J. (2019). Recent Advances in the Development of Neuroprotective Agents and Therapeutic Targets in the Treatment of Cerebral Ischemia. Eur. J. Med. Chem. 162, 132–146. 10.1016/j.ejmech.2018.11.014 30445263

[B93] Ma Y. Wang J. Wang Y. Yang G. Y. (2017). The Biphasic Function of Microglia in Ischemic Stroke. Prog. Neurobiol. 157, 247–272. 10.1016/j.pneurobio.2016.01.005 26851161

[B94] Mahjoob M. Stochaj U. (2021). Curcumin Nanoformulations to Combat Aging-Related Diseases. Ageing Res. Rev. 69, 101364. 10.1016/j.arr.2021.101364 34000462

[B95] Mahmood K. Zia K. M. Zuber M. Salman M. Anjum M. N. (2015). Recent Developments in Curcumin and Curcumin Based Polymeric Materials for Biomedical Applications: A Review. Int. J. Biol. Macromol 81, 877–890. 10.1016/j.ijbiomac.2015.09.026 26391597

[B96] Mandal M. Jaiswal P. Mishra A. (2020). Role of Curcumin and its Nanoformulations in Neurotherapeutics: A Comprehensive Review. J. Biochem. Mol. Toxicol. 34, e22478. 10.1002/jbt.22478 32124518

[B97] Marlier Q. Verteneuil S. Vandenbosch R. Malgrange B. (2015). Mechanisms and Functional Significance of Stroke-Induced Neurogenesis. Front. Neurosci. 9, 458. 10.3389/fnins.2015.00458 26696816 PMC4672088

[B98] Masuelli L. Benvenuto M. Di Stefano E. Mattera R. Fantini M. De Feudis G. (2017). Curcumin Blocks Autophagy and Activates Apoptosis of Malignant Mesothelioma Cell Lines and Increases the Survival of Mice Intraperitoneally Transplanted with a Malignant Mesothelioma Cell Line. Oncotarget 8, 34405–34422. 10.18632/oncotarget.14907 28159921 PMC5470978

[B99] Miao Y. Zhao S. Gao Y. Wang R. Wu Q. Wu H. (2016). Curcumin Pretreatment Attenuates Inflammation and Mitochondrial Dysfunction in Experimental Stroke: The Possible Role of Sirt1 Signaling. Brain Res. Bull. 121, 9–15. 10.1016/j.brainresbull.2015.11.019 26639783

[B100] Mitsios N. Gaffney J. Krupinski J. Mathias R. Wang Q. Hayward S. (2007). Expression of Signaling Molecules Associated with Apoptosis in Human Ischemic Stroke Tissue. Cell Biochem Biophys 47, 73–86. 10.1385/cbb:47:1:73 17406061

[B101] Mizushima N. Komatsu M. (2011). Autophagy: Renovation of Cells and Tissues. Cell 147, 728–741. 10.1016/j.cell.2011.10.026 22078875

[B102] Mizushima N. Levine B. Cuervo A. M. Klionsky D. J. (2008). Autophagy Fights Disease through Cellular Self-Digestion. Nature 451, 1069–1075. 10.1038/nature06639 18305538 PMC2670399

[B103] Mo Y. Yue E. Shi N. Liu K. (2021). The Protective Effects of Curcumin in Cerebral Ischemia and Reperfusion Injury through PKC-θ Signaling. Cell Cycle 20, 550–560. 10.1080/15384101.2021.1889188 33618616 PMC8018382

[B104] Mohajeri M. Sadeghizadeh M. Najafi F. Javan M. (2015). Polymerized Nano-Curcumin Attenuates Neurological Symptoms in EAE Model of Multiple Sclerosis through Down Regulation of Inflammatory and Oxidative Processes and Enhancing Neuroprotection and Myelin Repair. Neuropharmacology 99, 156–167. 10.1016/j.neuropharm.2015.07.013 26211978

[B105] Mondal N. K. Behera J. Kelly K. E. George A. K. Tyagi P. K. Tyagi N. (2019). Tetrahydrocurcumin Epigenetically Mitigates Mitochondrial Dysfunction in Brain Vasculature during Ischemic Stroke. Neurochem. Int. 122, 120–138. 10.1016/j.neuint.2018.11.015 30472160 PMC6666268

[B106] Moretti A. Ferrari F. Villa R. F. (2015). Pharmacological Therapy of Acute Ischaemic Stroke: Achievements and Problems. Pharmacol. Ther. 153, 79–89. 10.1016/j.pharmthera.2015.06.004 26079382

[B107] Mukherjee A. Sarkar S. Jana S. Swarnakar S. Das N. (2019). Neuro-protective Role of Nanocapsulated Curcumin against Cerebral Ischemia-Reperfusion Induced Oxidative Injury. Brain Res. 1704, 164–173. 10.1016/j.brainres.2018.10.016 30326199

[B108] Namgyal D. Ali S. Hussain M. D. Kazi M. Ahmad A. Sarwat M. (2021). Curcumin Ameliorates the Cd-Induced Anxiety-like Behavior in Mice by Regulating Oxidative Stress and Neuro-Inflammatory Proteins in the Prefrontal Cortex Region of the Brain. Antioxidants (Basel) 10, 1710. 10.3390/antiox10111710 34829581 PMC8614802

[B109] Nebrisi E. E. (2021). Neuroprotective Activities of Curcumin in Parkinson's Disease: A Review of the Literature. Int. J. Mol. Sci. 22, 11248. 10.3390/ijms222011248 34681908 PMC8537234

[B110] Notarbartolo M. Poma P. Perri D. Dusonchet L. Cervello M. D'alessandro N. (2005). Antitumor Effects of Curcumin, Alone or in Combination with Cisplatin or Doxorubicin, on Human Hepatic Cancer Cells. Analysis of Their Possible Relationship to Changes in NF-kB Activation Levels and in IAP Gene Expression. Cancer Lett. 224, 53–65. 10.1016/j.canlet.2004.10.051 15911101

[B111] Obermeier B. Daneman R. Ransohoff R. M. (2013). Development, Maintenance and Disruption of the Blood-Brain Barrier. Nat. Med. 19, 1584–1596. 10.1038/nm.3407 24309662 PMC4080800

[B112] Ojha R. P. Rastogi M. Devi B. P. Agrawal A. Dubey G. P. (2012). Neuroprotective Effect of Curcuminoids against Inflammation-Mediated Dopaminergic Neurodegeneration in the MPTP Model of Parkinson's Disease. J. Neuroimmune Pharmacol. 7, 609–618. 10.1007/s11481-012-9363-2 22527634

[B113] Olmez I. Ozyurt H. (2012). Reactive Oxygen Species and Ischemic Cerebrovascular Disease. Neurochem. Int. 60, 208–212. 10.1016/j.neuint.2011.11.009 22122807

[B114] Orellana-Urzúa S. Rojas I. Líbano L. Rodrigo R. (2020). Pathophysiology of Ischemic Stroke: Role of Oxidative Stress. Curr. Pharm. Des. 26, 4246–4260. 10.2174/1381612826666200708133912 32640953

[B115] Ovbiagele B. (2008). Potential Role of Curcumin in Stroke Prevention. Expert Rev. Neurother 8, 1175–1176. 10.1586/14737175.8.8.1175 18671659

[B116] Pan J. Konstas A. A. Bateman B. Ortolano G. A. Pile-Spellman J. (2007). Reperfusion Injury Following Cerebral Ischemia: Pathophysiology, MR Imaging, and Potential Therapies. Neuroradiology 49, 93–102. 10.1007/s00234-006-0183-z 17177065 PMC1786189

[B117] Peng T. I. Jou M. J. (2010). Oxidative Stress Caused by Mitochondrial Calcium Overload. Ann. N. Y Acad. Sci. 1201, 183–188. 10.1111/j.1749-6632.2010.05634.x 20649555

[B118] Pu Y. Zhang H. Wang P. Zhao Y. Li Q. Wei X. (2013). Dietary Curcumin Ameliorates Aging-Related Cerebrovascular Dysfunction through the AMPK/uncoupling Protein 2 Pathway. Cell Physiol Biochem 32, 1167–1177. 10.1159/000354516 24335167

[B119] Radak D. Katsiki N. Resanovic I. Jovanovic A. Sudar-Milovanovic E. Zafirovic S. (2017). Apoptosis and Acute Brain Ischemia in Ischemic Stroke. Curr. Vasc. Pharmacol. 15, 115–122. 10.2174/1570161115666161104095522 27823556

[B120] Ramaholimihaso T. Bouazzaoui F. Kaladjian A. (2020). Curcumin in Depression: Potential Mechanisms of Action and Current Evidence-A Narrative Review. Front. Psychiatry 11, 572533. 10.3389/fpsyt.2020.572533 33329109 PMC7728608

[B121] Rami A. Bechmann I. Stehle J. H. (2008). Exploiting Endogenous Anti-apoptotic Proteins for Novel Therapeutic Strategies in Cerebral Ischemia. Prog. Neurobiol. 85, 273–296. 10.1016/j.pneurobio.2008.04.003 18511172

[B122] Ran Y. Su W. Gao F. Ding Z. Yang S. Ye L. (2021). Curcumin Ameliorates White Matter Injury after Ischemic Stroke by Inhibiting Microglia/Macrophage Pyroptosis through NF-Κb Suppression and NLRP3 Inflammasome Inhibition. Oxid Med. Cel Longev 2021, 1552127. 10.1155/2021/1552127 PMC8497115 34630845

[B123] Rathore P. Dohare P. Varma S. Ray A. Sharma U. Jagannathan N. R. (2008). Curcuma Oil: Reduces Early Accumulation of Oxidative Product and Is Anti-apoptogenic in Transient Focal Ischemia in Rat Brain. Neurochem. Res. 33, 1672–1682. 10.1007/s11064-007-9515-6 17955367

[B124] Rottenberg H. Hoek J. B. (2017). The Path from Mitochondrial ROS to Aging Runs through the Mitochondrial Permeability Transition Pore. Aging Cell 16, 943–955. 10.1111/acel.12650 28758328 PMC5595682

[B125] Saleh D. O. Nasr M. Hassan A. El-Awdan S. A. Abdel Jaleel G. A. (2022). Curcumin Nanoemulsion Ameliorates Brain Injury in Diabetic Rats. J. Food Biochem., e14104. 10.1111/jfbc.14104 35098560

[B126] Seo S. U. Kim T. H. Kim D. E. Min K. J. Kwon T. K. (2017). NOX4-mediated ROS Production Induces Apoptotic Cell Death via Down-Regulation of C-FLIP and Mcl-1 Expression in Combined Treatment with Thioridazine and Curcumin. Redox Biol. 13, 608–622. 10.1016/j.redox.2017.07.017 28806703 PMC5554966

[B127] Shah F. A. Gim S. A. Sung J. H. Jeon S. J. Kim M. O. Koh P. O. (2016). Identification of Proteins Regulated by Curcumin in Cerebral Ischemia. J. Surg. Res. 201, 141–148. 10.1016/j.jss.2015.10.025 26850195

[B128] Shah F. A. Park D. J. Gim S. A. Koh P. O. (2015). Curcumin Treatment Recovery the Decrease of Protein Phosphatase 2A Subunit B Induced by Focal Cerebral Ischemia in Sprague-Dawley Rats. Lab. Anim. Res. 31, 134–138. 10.5625/lar.2015.31.3.134 26472966 PMC4602080

[B129] Shao A. Lin D. Wang L. Tu S. Lenahan C. Zhang J. (2020). Oxidative Stress at the Crossroads of Aging, Stroke and Depression. Aging Dis. 11, 1537–1566. 10.14336/AD.2020.0225 33269106 PMC7673857

[B130] Shi K. Tian D. C. Li Z. G. Ducruet A. F. Lawton M. T. Shi F. D. (2019). Global Brain Inflammation in Stroke. Lancet Neurol. 18, 1058–1066. 10.1016/S1474-4422(19)30078-X 31296369

[B131] Shukla P. K. Khanna V. K. Ali M. M. Khan M. Y. Srimal R. C. (2008). Anti-ischemic Effect of Curcumin in Rat Brain. Neurochem. Res. 33, 1036–1043. 10.1007/s11064-007-9547-y 18204970

[B132] Smith C. M. Chen Y. Sullivan M. L. Kochanek P. M. Clark R. S. (2011). Autophagy in Acute Brain Injury: Feast, Famine, or Folly? Neurobiol. Dis. 43, 52–59. 10.1016/j.nbd.2010.09.014 20883784 PMC3046326

[B133] Sorce S. Krause K. H. Jaquet V. (2012). Targeting NOX Enzymes in the central Nervous System: Therapeutic Opportunities. Cell Mol Life Sci 69, 2387–2407. 10.1007/s00018-012-1014-5 22643836 PMC11114708

[B134] Srivastava P. Dhuriya Y. K. Gupta R. Shukla R. K. Yadav R. S. Dwivedi H. N. (2018). Protective Effect of Curcumin by Modulating BDNF/DARPP32/CREB in Arsenic-Induced Alterations in Dopaminergic Signaling in Rat Corpus Striatum. Mol. Neurobiol. 55, 445–461. 10.1007/s12035-016-0288-2 27966075

[B135] Su X. T. Wang L. Ma S. M. Cao Y. Yang N. N. Lin L. L. (2020). Mechanisms of Acupuncture in the Regulation of Oxidative Stress in Treating Ischemic Stroke. Oxid Med. Cel Longev 2020, 7875396. 10.1155/2020/7875396 PMC7644298 33178387

[B136] Subedi L. Gaire B. P. (2021). Neuroprotective Effects of Curcumin in Cerebral Ischemia: Cellular and Molecular Mechanisms. ACS Chem. Neurosci. 12, 2562–2572. 10.1021/acschemneuro.1c00153 34251185

[B137] Sun G. Miao Z. Ye Y. Zhao P. Fan L. Bao Z. (2020). Curcumin Alleviates Neuroinflammation, Enhances Hippocampal Neurogenesis, and Improves Spatial Memory after Traumatic Brain Injury. Brain Res. Bull. 162, 84–93. 10.1016/j.brainresbull.2020.05.009 32502596

[B138] Sun Y. Zhang T. Zhang Y. Li J. Jin L. Sun Y. (2018). Ischemic Postconditioning Alleviates Cerebral Ischemia-Reperfusion Injury through Activating Autophagy during Early Reperfusion in Rats. Neurochem. Res. 43, 1826–1840. 10.1007/s11064-018-2599-3 30046966 PMC6096887

[B139] Torres-Cuevas I. Corral-Debrinski M. Gressens P. (2019). Brain Oxidative Damage in Murine Models of Neonatal Hypoxia/ischemia and Reoxygenation. Free Radic. Biol. Med. 142, 3–15. 10.1016/j.freeradbiomed.2019.06.011 31226400

[B140] Tu X. K. Yang W. Z. Chen J. P. Chen Y. Ouyang L. Q. Xu Y. C. (2014). Curcumin Inhibits TLR2/4-NF-Κb Signaling Pathway and Attenuates Brain Damage in Permanent Focal Cerebral Ischemia in Rats. Inflammation 37, 1544–1551. 10.1007/s10753-014-9881-6 24723245

[B141] Tyagi N. Qipshidze N. Munjal C. Vacek J. C. Metreveli N. Givvimani S. (2012). Tetrahydrocurcumin Ameliorates Homocysteinylated Cytochrome-C Mediated Autophagy in Hyperhomocysteinemia Mice after Cerebral Ischemia. J. Mol. Neurosci. 47, 128–138. 10.1007/s12031-011-9695-z 22212488 PMC3609416

[B142] Ueda H. Fujita R. (2004). Cell Death Mode Switch from Necrosis to Apoptosis in Brain. Biol. Pharm. Bull. 27, 950–955. 10.1248/bpb.27.950 15256720

[B143] Ułamek-Kozioł M. Czuczwar S. J. Januszewski S. Pluta R. (2020). Substantiation for the Use of Curcumin during the Development of Neurodegeneration after Brain Ischemia. Int. J. Mol. Sci. 21, 517. 10.3390/ijms21020517 PMC7014172 31947633

[B144] Ullah F. Liang A. Rangel A. Gyengesi E. Niedermayer G. Münch G. (2017). High Bioavailability Curcumin: an Anti-inflammatory and Neurosupportive Bioactive Nutrient for Neurodegenerative Diseases Characterized by Chronic Neuroinflammation. Arch. Toxicol. 91, 1623–1634. 10.1007/s00204-017-1939-4 28204864

[B145] Uzdensky A. B. (2019). Apoptosis Regulation in the Penumbra after Ischemic Stroke: Expression of Pro- and Antiapoptotic Proteins. Apoptosis 24, 687–702. 10.1007/s10495-019-01556-6 31256300

[B146] Vajragupta O. Boonchoong P. Watanabe H. Tohda M. Kummasud N. Sumanont Y. (2003). Manganese Complexes of Curcumin and its Derivatives: Evaluation for the Radical Scavenging Ability and Neuroprotective Activity. Free Radic. Biol. Med. 35, 1632–1644. 10.1016/j.freeradbiomed.2003.09.011 14680686

[B147] Wakade C. King M. D. Laird M. D. Alleyne C. H. Jr. Dhandapani K. M. (2009). Curcumin Attenuates Vascular Inflammation and Cerebral Vasospasm after Subarachnoid Hemorrhage in Mice. Antioxid. Redox Signal. 11, 35–45. 10.1089/ars.2008.2056 18752423

[B148] Walker B. C. Mittal S. (2020). Antitumor Activity of Curcumin in Glioblastoma. Int. J. Mol. Sci. 21, 9435. 10.3390/ijms21249435 PMC7763573 33322413

[B149] Wang C. Yang Y. H. Zhou L. Ding X. L. Meng Y. C. Han K. (2020). Curcumin Alleviates OGD/R-induced PC12 Cell Damage via Repressing CCL3 and Inactivating TLR4/MyD88/MAPK/NF-κB to Suppress Inflammation and Apoptosis. J. Pharm. Pharmacol. 72, 1176–1185. 10.1111/jphp.13293 32436614

[B150] Wang P. Shao B. Z. Deng Z. Chen S. Yue Z. Miao C. Y. (2018). Autophagy in Ischemic Stroke. Prog. Neurobiol. 163-164, 98–117. 10.1016/j.pneurobio.2018.01.001 29331396

[B151] Wang Q. Sun A. Y. Simonyi A. Jensen M. D. Shelat P. B. Rottinghaus G. E. (2005). Neuroprotective Mechanisms of Curcumin against Cerebral Ischemia-Induced Neuronal Apoptosis and Behavioral Deficits. J. Neurosci. Res. 82, 138–148. 10.1002/jnr.20610 16075466

[B152] Wang W. Xu J. (2020). Curcumin Attenuates Cerebral Ischemia-Reperfusion Injury through Regulating Mitophagy and Preserving Mitochondrial Function. Curr. Neurovasc Res. 17, 113–122. 10.2174/1567202617666200225122620 32096742

[B153] Wang X. Fang Y. Huang Q. Xu P. Lenahan C. Lu J. (2021). An Updated Review of Autophagy in Ischemic Stroke: From Mechanisms to Therapies. Exp. Neurol. 340, 113684. 10.1016/j.expneurol.2021.113684 33676918

[B154] Wang Y. Luo J. Li S. Y. (2019a). Nano-Curcumin Simultaneously Protects the Blood-Brain Barrier and Reduces M1 Microglial Activation during Cerebral Ischemia-Reperfusion Injury. ACS Appl. Mater. Inter. 11, 3763–3770. 10.1021/acsami.8b20594 30618231

[B155] Wang Y. Zhang J. H. Sheng J. Shao A. (2019b). Immunoreactive Cells after Cerebral Ischemia. Front. Immunol. 10, 2781. 10.3389/fimmu.2019.02781 31849964 PMC6902047

[B156] Wang Y. Zhang Y. Yang L. Yuan J. Jia J. Yang S. (2019c). SOD2 Mediates Curcumin-Induced Protection against Oxygen-Glucose Deprivation/Reoxygenation Injury in HT22 Cells. Evid. Based Complement. Alternat Med. 2019, 2160642. 10.1155/2019/2160642 31662771 PMC6791267

[B157] Wang Y. F. Gu Y. T. Qin G. H. Zhong L. Meng Y. N. (2013). Curcumin Ameliorates the Permeability of the Blood-Brain Barrier during Hypoxia by Upregulating Heme Oxygenase-1 Expression in Brain Microvascular Endothelial Cells. J. Mol. Neurosci. 51, 344–351. 10.1007/s12031-013-9989-4 23494637

[B158] Wang Z. Ren W. Zhao F. Han Y. Liu C. Jia K. (2020). Curcumin Amends Ca2+ Dysregulation in Microglia by Suppressing the Activation of P2X7 Receptor. Mol. Cel Biochem 465, 65–73. 10.1007/s11010-019-03668-8 31894530

[B159] Wardlaw J. M. Murray V. Berge E. Del Zoppo G. J. (2014). Thrombolysis for Acute Ischaemic Stroke. Cochrane Database Syst. Rev. 2014, Cd000213. 10.1002/14651858.CD000213 PMC4153726 25072528

[B160] Wicha P. Tocharus J. Janyou A. Jittiwat J. Chaichompoo W. Suksamrarn A. (2020). Hexahydrocurcumin Alleviated Blood-Brain Barrier Dysfunction in Cerebral Ischemia/reperfusion Rats. Pharmacol. Rep. 72, 659–671. 10.1007/s43440-019-00050-9 32048258

[B161] Wicha P. Tocharus J. Janyou A. Jittiwat J. Changtam C. Suksamrarn A. (2017). Hexahydrocurcumin Protects against Cerebral Ischemia/reperfusion Injury, Attenuates Inflammation, and Improves Antioxidant Defenses in a Rat Stroke Model. PLoS One 12, e0189211. 10.1371/journal.pone.0189211 29220411 PMC5722321

[B162] Wolf M. S. Bayır H. Kochanek P. M. Clark R. S. B. (2019). The Role of Autophagy in Acute Brain Injury: A State of Flux? Neurobiol. Dis. 122, 9–15. 10.1016/j.nbd.2018.04.018 29704549 PMC6203674

[B163] Wu J. Li Q. Wang X. Yu S. Li L. Wu X. (2013). Neuroprotection by Curcumin in Ischemic Brain Injury Involves the Akt/Nrf2 Pathway. PLoS One 8, e59843. 10.1371/journal.pone.0059843 23555802 PMC3610879

[B164] Wu J. X. Zhang L. Y. Chen Y. L. Yu S. S. Zhao Y. Zhao J. (2015). Curcumin Pretreatment and post-treatment Both Improve the Antioxidative Ability of Neurons with Oxygen-Glucose Deprivation. Neural Regen. Res. 10, 481–489. 10.4103/1673-5374.153700 25878600 PMC4396114

[B165] Wu L. Jiang C. Kang Y. Dai Y. Fang W. Huang P. (2020). Curcumin Exerts Protective Effects against Hypoxia-reoxygenation I-njury via the E-nhancement of A-purinic/apyrimidinic E-ndonuclease 1 in SH-SY5Y C-ells: Involvement of the PI3K/AKT P-athway. Int. J. Mol. Med. 45, 993–1004. 10.3892/ijmm.2020.4483 32124937 PMC7053876

[B166] Wu S. Guo T. Qi W. Li Y. Gu J. Liu C. (2021). Curcumin Ameliorates Ischemic Stroke Injury in Rats by Protecting the Integrity of the Blood-Brain Barrier. Exp. Ther. Med. 22, 783. 10.3892/etm.2021.10215 34055082 PMC8145684

[B167] Xia M. Ye Z. Shi Y. Zhou L. Hua Y. (2018). Curcumin Improves Diabetes Mellitus-associated C-erebral I-nfarction by I-ncreasing the E-xpression of GLUT1 and GLUT3. Mol. Med. Rep. 17, 1963–1969. 10.3892/mmr.2017.8085 29257220

[B168] Xie C. J. Gu A. P. Cai J. Wu Y. Chen R. C. (2018). Curcumin Protects Neural Cells against Ischemic Injury in N2a Cells and Mouse Brain with Ischemic Stroke. Brain Behav. 8, e00921. 10.1002/brb3.921 29484272 PMC5822585

[B169] Xiong X. Y. Liu L. Yang Q. W. (2016). Functions and Mechanisms of Microglia/macrophages in Neuroinflammation and Neurogenesis after Stroke. Prog. Neurobiol. 142, 23–44. 10.1016/j.pneurobio.2016.05.001 27166859

[B170] Xu H. Nie B. Liu L. Zhang C. Zhang Z. Xu M. (2019). Curcumin Prevents Brain Damage and Cognitive Dysfunction during Ischemic-Reperfusion through the Regulation of miR-7-5p. Curr. Neurovasc Res. 16, 441–454. 10.2174/1567202616666191029113633 31660818

[B171] Xu J. Kong X. Xiu H. Dou Y. Wu Z. Sun P. (2018). Combination of Curcumin and Vagus Nerve Stimulation Attenuates Cerebral Ischemia/reperfusion Injury-Induced Behavioral Deficits. Biomed. Pharmacother. 103, 614–620. 10.1016/j.biopha.2018.04.069 29677548

[B172] Xu L. Ding L. Su Y. Shao R. Liu J. Huang Y. (2019). Neuroprotective Effects of Curcumin against Rats with Focal Cerebral Ischemia-Reperfusion Injury. Int. J. Mol. Med. 43, 1879–1887. 10.3892/ijmm.2019.4094 30816425

[B173] Xu Y. Ku B. Tie L. Yao H. Jiang W. Ma X. (2006). Curcumin Reverses the Effects of Chronic Stress on Behavior, the HPA axis, BDNF Expression and Phosphorylation of CREB. Brain Res. 1122, 56–64. 10.1016/j.brainres.2006.09.009 17022948

[B174] Xue Y. Nie D. Wang L. J. Qiu H. C. Ma L. Dong M. X. (2021). Microglial Polarization: Novel Therapeutic Strategy against Ischemic Stroke. Aging Dis. 12, 466–479. 10.14336/AD.2020.0701 33815877 PMC7990355

[B175] Yan D. Wang N. Yao J. Wu X. Yuan J. Yan H. (2022). Curcumin Attenuates the PERK-eIF2α Signaling to Relieve Acrylamide-Induced Neurotoxicity in SH-SY5Y Neuroblastoma Cells. Neurochem. Res. 47, 1037–1048. 10.1007/s11064-021-03504-w 35037165

[B176] Yang B. Luo G. Zhang C. Feng L. Luo X. Gan L. (2020). Curcumin Protects Rat Hippocampal Neurons against Pseudorabies Virus by Regulating the BDNF/TrkB Pathway. Sci. Rep. 10, 22204. 10.1038/s41598-020-78903-0 33335121 PMC7746732

[B177] Yang J. (2019). The Role of Reactive Oxygen Species in Angiogenesis and Preventing Tissue Injury after Brain Ischemia. Microvasc. Res. 123, 62–67. 10.1016/j.mvr.2018.12.005 30594490

[B178] Yang X. Song D. Chen L. Xiao H. Ma X. Jiang Q. (2021). Curcumin Promotes Neurogenesis of Hippocampal Dentate Gyrus via Wnt/β-Catenin Signal Pathway Following Cerebral Ischemia in Mice. Brain Res. 1751, 147197. 10.1016/j.brainres.2020.147197 33160958

[B179] Yavarpour-Bali H. Ghasemi-Kasman M. Pirzadeh M. (2019). Curcumin-loaded Nanoparticles: a Novel Therapeutic Strategy in Treatment of central Nervous System Disorders. Int. J. Nanomedicine 14, 4449–4460. 10.2147/IJN.S208332 31417253 PMC6592058

[B180] Youssef M. I. Ma J. Chen Z. Hu W. W. (2021). Potential Therapeutic Agents for Ischemic white Matter Damage. Neurochem. Int. 149, 105116. 10.1016/j.neuint.2021.105116 34229025

[B181] Yu L. Tao J. Zhao Q. Xu C. Zhang Q. (2020). Confirmation of Potential Neuroprotective Effects of Natural Bioactive Compounds from Traditional Medicinal Herbs in Cerebral Ischemia Treatment. J. Integr. Neurosci. 19, 373–384. 10.31083/j.jin.2020.02.63 32706202

[B182] Yuan J. Botchway B. O. A. Zhang Y. Tan X. Wang X. Liu X. (2019). Curcumin Can Improve Spinal Cord Injury by Inhibiting TGF-β-SOX9 Signaling Pathway. Cell Mol Neurobiol 39, 569–575. 10.1007/s10571-019-00671-x 30915623 PMC11462994

[B183] Zhang N. Wei G. Ye J. Yang L. Hong Y. Liu G. (2017). Effect of Curcumin on Acute Spinal Cord Injury in Mice via Inhibition of Inflammation and TAK1 Pathway. Pharmacol. Rep. 69, 1001–1006. 10.1016/j.pharep.2017.02.012 28941865

[B184] Zhang T. Chen X. Qu Y. Ding Y. (2021). Curcumin Alleviates Oxygen-Glucose-Deprivation/Reperfusion-Induced Oxidative Damage by Regulating miR-1287-5p/LONP2 Axis in SH-Sy5y Cells. Anal. Cel Pathol (Amst) 2021, 5548706. 10.1155/2021/5548706 PMC8476263 34589382

[B185] Zhang Y. Fang M. Sun Y. Zhang T. Shi N. Li J. (2018). Curcumin Attenuates Cerebral Ischemia Injury in Sprague-Dawley Rats and PC12 Cells by Suppressing Overactivated Autophagy. J. Photochem. Photobiol. B 184, 1–6. 10.1016/j.jphotobiol.2018.05.010 29777940

[B186] Zhang Y. Yan Y. Cao Y. Yang Y. Zhao Q. Jing R. (2017b). Potential Therapeutic and Protective Effect of Curcumin against Stroke in the Male Albino Stroke-Induced Model Rats. Life Sci. 183, 45–49. 10.1016/j.lfs.2017.06.023 28663065

[B187] Zhao S. C. Ma L. S. Chu Z. H. Xu H. Wu W. Q. Liu F. (2017). Regulation of Microglial Activation in Stroke. Acta Pharmacol. Sin 38, 445–458. 10.1038/aps.2016.162 28260801 PMC5386316

[B188] Zheng Z. Zhao H. Steinberg G. K. Yenari M. A. (2003). Cellular and Molecular Events Underlying Ischemia-Induced Neuronal Apoptosis. Drug News Perspect. 16, 497–503. 10.1358/dnp.2003.16.8.829348 14668947

[B189] Zhou H. Beevers C. S. Huang S. (2011). The Targets of Curcumin. Curr. Drug Targets 12, 332–347. 10.2174/138945011794815356 20955148 PMC3025067

[B190] Zhou H. Y. Sun Y. Y. Chang P. Huang H. C. (2022). Curcumin Inhibits Cell Damage and Apoptosis Caused by Thapsigargin-Induced Endoplasmic Reticulum Stress Involving the Recovery of Mitochondrial Function Mediated by Mitofusin-2. Neurotox Res. 10.1007/s12640-022-00481-y 35192145

[B191] Zhou J. Wu N. Lin L. (2020). Curcumin Suppresses Apoptosis and Inflammation in Hypoxia/Reperfusion-Exposed Neurons via Wnt Signaling Pathway. Med. Sci. Monit. 26, e920445. 10.12659/MSM.920445 32107363 PMC7061587

[B192] Zhou Y. Zhang S. Fan X. (2021). Role of Polyphenols as Antioxidant Supplementation in Ischemic Stroke. Oxid Med. Cel Longev 2021, 5471347. 10.1155/2021/5471347 PMC8253632 34257802

[B193] Zhu H. Fan Y. Sun H. Chen L. Man X. (2017). Curcumin Inhibits Endoplasmic Reticulum Stress Induced by Cerebral Ischemia-Reperfusion Injury in Rats. Exp. Ther. Med. 14, 4047–4052. 10.3892/etm.2017.5040 29067098 PMC5647704

[B194] Zhu T. Wang L. Feng Y. Sun G. Sun X. (2021). Classical Active Ingredients and Extracts of Chinese Herbal Medicines: Pharmacokinetics, Pharmacodynamics, and Molecular Mechanisms for Ischemic Stroke. Oxid Med. Cel Longev 2021, 8868941. 10.1155/2021/8868941 PMC7984881 33791075

[B195] Živančević K. Lović D. Andjus P. R. Radenović L. (2021). “Neuroinflammation in Post-Ischemic Brain,” in Cerebral Ischemia. Editor Pluta R. (Brisbane (AU): Exon Publications). 34905311

