# Supplementary material for: Mechanisms Underlying Curcumin-Induced Neuroprotection in Cerebral Ischemia
Source: Front Pharmacol. 2022 Apr 26;13:893118. doi: 10.3389/fphar.2022.893118 (PMC9090137; doi:10.3389/fphar.2022.893118)
Supplement: Supplementary file 1 [file DataSheet1.docx]

**Supplementary Table 1 Basic research of curcumin treatment in ischemic stroke in the past decade.**

| **Ref.** | **Species** | **Model** | **Drug** | **Approach** | **Dosage** | **Interval** | **Outcome** | **Mechanism** |
| --- | --- | --- | --- | --- | --- | --- | --- | --- |
| (Saleh et al., 2022) | Wistar rats | Diabetes and MCAO/R | Curcumin nanoemulsion | Oral administration. | 50 and 100 mg/kg | Once daily for 2 weeks. | Attenuate neurological deficit score and imbalance of redox homeostasis. | Reduce the expression of TNF, IL-1β, COX-2, cleaved caspase-3, and NF-κB, restore the expression of glucose transporter 1 proteins. |
| (Ran et al., 2021) | C57BL/6J Mice | MCAO/R | Curcumin | Intraperitoneal administration. | 150 mg/kg | Once daily for 7 days. | Reduce white matter damage, improve functional outcomes, and attenuate microglial pyroptosis. | Suppression of the NF-κB/NLRP3 signaling pathway. |
| (Zhang et al., 2021) | SH-SY5Y cells | OGD/R | Curcumin | Add to the culture medium. | 25 μm/l | Pretreatment for 24 h. | Alleviate cell damage, inhibit apoptosis and ROS generation. | Regulate the miR-1287-5p/LONP2 axis. |
| (Huang et al., 2021) | Rat cortical neurons; SD rats | OGD, MCAO/R | Curcumin | Not mentioned. | Not mentioned | Not mentioned. | Promote neuronal proliferation and inhibit neuronal pyroptosis. Attenuate cerebral infarction. | Inhibit NLRP1-dependent neuronal pyroptosis by suppressing the p38 MAPK pathway |
| (Wu et al., 2021) | SD rats | MCAO/R | Curcumin | Intraperitoneal administration. | 300 mg/kg | 30 min prior to surgery. | Improve neurological scores, decrease infarct size. | Protect BBB integrity and synaptic remodeling, inhibit inflammatory responses. |
| (Li et al., 2021) | Wistar rats | BCCAO | Triblock copolymer nanomicelles loaded with curcumin | Oral administration. | 80mg/kg | For 14 days; 24 h after the last gavage, the stroke is induced. | Attenuate neuronal damage and degeneration through antioxidant and anti-inflammatory effects. | Reduce the inflammatory signal transduction pathway including Il-1β, IL-6, TNF-α, and NF-ĸB, and improve lipid peroxidation |
| (Mo et al., 2021) | Rat PC12 cells; SD rats | OGD/R; MCAO/R | Curcumin | Intraperitoneal administration. | 150 mg/kg | Cell treat with different concentrations of curcumin; rat pretreat twice (24 h and 1 h) before surgery. | Attenuate neurologic dysfunction, preserve BBB integrity. | Regulate PKC-θ, which in turn mediate Ca^2+^ concentrations that affect BBB integrity. |
| (Yang et al., 2021) | C57BL/6 mice | BCCAO | Curcumin | Intraperitoneal administration. | 50, 100 mg/kg | Once daily for 7 days, starting at 3 h after surgery. | Alleviate cognitive deficits; dose-dependently increase the proliferation of NSCs and hippocampal neurogenesis. | Increase the expression of proteins involved in neurogenesis (including Ngn2, Pax6 and NeuroD 1) and the Wnt/β-catenin signaling pathway. |
| (He et al., 2020) | SD rats | MCAO/R | Curcumin-laden exosomes | Intravenous administration. | 10 μg/mL | Inject at 2 h after surgery. | Improve neurological deficits, and reduce infarct size. | Reduce ROS accumulation, alleviate BBB damage, and suppress mitochondria-mediated neuronal apoptosis. |
| (Wang et al., 2020a) | PC12 cells | OGD/R | Curcumin | Add to the culture medium | 20 μm/l | Not mentioned. | Alleviate the decrease of cell viability, and the increase of cell apoptosis. | Repress CCL3 and inactivate TLR4/MyD88/MAPK/NF-κB to suppress inflammation and apoptosis |
| (Kamali Dolatabadi et al., 2019) | SD rats | GCI | Curcumin | Oral administration. | 50, 100 mg/kg | Once daily for 7 or 28 days, starting at 16-24 h after surgery. | Improve memory and neurological deficits. | Restore irregular neuronal distribution in the CA1 region in a time-dependent manner. |
| (Wu et al., 2020) | SH‑SY5Y cells | OGD/R | Curcumin | Add to the culture medium | Not mentioned | For 24 h immediately after 1 h of OGD exposure during reperfusion | Increase cell viability, reduce apoptosis and oxidative stress. | Enhance the APE1 level and activity, promote PI3K/AKT pathway activation. |
| (Zhou et al., 2020) | Primary neonatal neurons | H/R | Curcumin | Add to the culture medium | 0.5, 1, 2, 4, and 8 μm/L | Pretreat neurons for 48 h prior to H/R. | Increase cell viability and inhibit apoptosis of neurons in a concentration-dependent manner. | Inhibit activation of the Wnt/JNK1 signaling pathway. |
| (Wang and Xu, 2020) | SD rats; Primary neurons | MCAO/R; OGD/R | Curcumin | Intraperitoneal administration; add to the culture medium | 100 mg/kg; 5µM | Once at the onset of reperfusion. | Decrease neurological deficit score, infarct volume, and morphological changes of neurons. | Regulate mitophagy and preserve mitochondrial function. |
| (Wicha et al., 2020) | Wistar rats | MCAO/R | Hexahydrocurcumin | Intraperitoneal administration. | 40 mg/kg | Once at the onset of reperfusion. | Reduce neurological scores, infarct volume, morphological changes, and brain water content. | Preserve the BBB by regulating TJPs, attenuating neutrophil infiltration, and reducing brain edema formation. |
| (Wang et al., 2019c) | HT22 neuronal cells | OGD/R | Curcumin | Add to the culture medium. | 10, 100 and 500 ng/ml | Once at the onset of OGD | Reduce the cell injury and apoptosis, attenuate damaged cell morphology. | Inhibit intracellular ROS and mitochondrial SOD accumulation, ameliorate intracellular SOD2, MMP, and mitochondrial complex I activity. |
| (Xu et al., 2019a) | SD rats; PC12 cells | MCAO/R; OGD/R | Curcumin | Intragastric administration; add to the culture medium. | 100 and 400 mg/kg; 10 and 20 µm/L | Once daily for 3 days, starting at 24 h after surgery; once after 24h reoxygenation. | Attenuate cell necrosis and apoptosis, inflammatory response and oxidative stress. | Regulation of the miR-7/RELA p65 axis. |
| (Hou et al., 2019) | PC12 cells | OGD/R | Curcumin | Add to the culture medium. | 1.25–20 μM | Once at the onset of reperfusion. | Decrease the death and apoptosis of cells. | Regulation of the reciprocal function between autophagy and HIF-1α. |
| (Xu et al., 2019b) | SD rats; primary astrocyte cells | MCAO/R; OGD/R | Curcumin | Intraperitoneal administration; add to the culture medium. | 100 and 300 mg/kg; 5, 10, 20 µm/l | 30 min prior to surgery and daily injection; 24 h prior to OGD. | Improve nerve damage symptoms and infarct volume, reduce brain water content, and relieve neuronal apoptosis. | Regulation of MEK/ERK/CREB pathway. |
| (Wang et al., 2019a) | Primary microvascular endothelial cells; C57BL/6J mice | MCAO/R | Curcumin-encapsulated nanoparticle | Add to the culture medium; intravenous administration. | 0.2 mg/ml; 25 mg/kg. | For 48 h, 3 h prior sacrifice. | Reduce the infarct size and improve function recovery. | Protect the BBB and reduce M1 microglial activation. |
| (Mondal et al., 2019) | C57BL/6J mice | MCAO/R | Tetrahydrocurcumin | Intraperitoneal administration. | 25mg/kg | Once daily for 3 days after 4 h of ischemia. | Improve neurological score and functional capacity. Ameliorate mitochondrial dysfunction in brain vasculature. | Abrogate hyper-methylation of TIMP-2 promoter and maintain the extracellular matrix integrity |
| (Mukherjee et al., 2019) | SD rats | BCCAO | Nanocapsulated curcumin | Oral administration. | 5 mg/kg | 24 h prior to surgery. | Maintain normal neuronal structure and function. | Ameliorate ROS-mediated oxidative damage and prevent neuronal apoptosis. |
| (Zhang et al., 2018) | SD rats | MCAO/R | Curcumin | Oral administration | 100 and 200 mg/kg | For 3 weeks after MCAO/R. | Attenuate neurological score, LDH activity, autophagy and apoptosis. | Decrease LC3 II/I protein and caspase 3 expression and increase p62 protein expression. |
| (Xu et al., 2018) | SD rats | MCAO/R | Combination of curcumin and vagus nerve stimulation | Intraperitoneal administration. | 50 mg/kg | For 5 days before surgery. | Restore behavioral deficits by inhibiting apoptosis and inflammatory response. | Regulation of AKT/ERK2 pathway. |
| (Lu et al., 2018) | Primary mouse cortical neurons | OGD/R | Curcumin | Add to the culture medium. | 0.25-1.0 mM | For 2-8 h. | Inhibite neuronal death and increase cell survival. | Regulation of flotillin-1 and ERK1/2 pathway. |
| (Xia et al., 2018) | SD rats | Diabetes and MCAO/R | Curcumin | Intragastric administration. | 40 mg/kg | Not mentioned. | Improve neurological deficits, reduce brain edema, and infarct volume | Reduce cell apoptosis. Increase the expression of GLUT1 and GLUT3. |
| (Huang et al., 2018a) | SD rats | MCAO/R | Curcumin | Intraperitoneal administration. | 200 mg/kg | Once at 30 min after MCAO/R. | Improve neurological function, and reduce infarct volume. | Attenuate autophagic activities through mediating the PI3K/Akt/mTOR pathway, while suppress an inflammatory reaction by regulating the TLR4/p38/MAPK pathway |
| (Wicha et al., 2017) | Wistar rats | MCAO/R | Hexahydrocurcumin | Intraperitoneal administration. | 10, 20 and 40 mg/kg | Once at the onset of reperfusion. | Improve neurological function, and reduce infarct volume. | Diminish oxidative stress, inflammation, and apoptosis |
| (Zhu et al., 2017) | Wistar rats | CIRI | Curcumin | Intraperitoneal administration. | 150 mg/kg | Once at 2 h prior to surgery | Attenuate neurological deficits. | Inhibit endoplasmic reticulum stress by decreasing GADD153 and caspase-12. |
| (Li et al., 2017) | Wistar rats | MCAO/R | Curcumin | Intraperitoneal administration. | 300 mg/kg | Once at 2 h prior to reperfusion. | Attenuate brain infarction size, brain edema, and neurological dysfunction. | Inflammation (NF-κB, ICAM-1 and MMP-9) and apoptosis (caspase-3)-related markers are downregulated. |
| (Liu et al., 2017) | C57BL/6 mice | dMCAO | Curcumin | Intraperitoneal administration. | 150 mg/kg | Immediately after ischemia and 24 h later. | Improve neurological function, reduce brain infarction. | Inhibit LPS and IFN-γ-induced M1 polarization; promote M2 microglial polarization and inhibit microglia-mediated pro-inflammatory responses. |
| (Zhang et al., 2017b) | Albino rats | MCAO/R | Curcumin | Not mentioned. | 25 mg/kg | Not mentioned. | Reduce brain edema. | Reduce the expression of IL-6, TNF-α, MMP, p53 and Bax. Increase the level of Bcl-2 and Sirt1. |
| (Jia et al., 2017) | SD rats | MCAO/R | Curcumin | Intraperitoneal administration. | 300 mg/kg | Once at 1 h after reperfusion. | Reduce infarct size and neurological deficiency. | Regulation of SP1/Prdx6 pathway. |
| (de Alcântara et al., 2017) | Wistar rats | MCAO/R | Curcumin | Orally administration. | 25 and 50 mg/kg | 1 h prior to surgery and 24 h later; 1h prior to surgery and once daily for 7 days. | Increase neuronal viability. | Increase the level f of DA, HVA, DOPAC and 5-HT, and attenuated the immunoreactivity for COX-2 and TNF-α. |
| (Lin et al., 2016) | ICR mice | BCCAO | Tetrahydrocurcumin | Intraperitoneal administration. | 5, 10 and 25 mg/kg | Once at 5 min after reperfusion. | Reduce neurological deficits, infarct area, and neuronal necrosis. | Exhibit a dose-dependent protective effect mediated by suppression of the ERK signaling pathway and a subsequent reduction in GRASP65 phosphorylation. |
| (Kalani et al., 2016) | C57BL/6J mice | MCAO/R | Curcumin -loaded embryonic stem cell exosomes | Intranasal administration. | 10 μl MESC-exocur | Twice a day for 7 days, starting within 1 h of ischemia. | Reduce infarct volume and cerebral edema, improve neurological function. | Reduce oxidative stress, vascular inflammation, and NMDAR1 expression, restored neurons, alleviate tight and adherent junctions |
| (Altinay et al., 2017) | Wistar rats | BCCAO | Curcumin | Orally and intraperitoneal administration. | 300 mg/kg | 21 days before surgery and once after surgery. | Improve neurological function, and reduce apoptosis. | Increase the enzyme activity of SOD, GPX, and CAT, decrease the activity of MDA, and reduce the concentrations of IL-6 and TNF-α. |
| (Liu et al., 2016) | SD rats | MCAO | Curcumin | Intranasal administration. | 300 mg/kg | Starting 1 h after stroke and continuing once daily for 7 days. | Reduce neurological deficits. | Activate Notch signaling pathway. |
| (Shah et al., 2016) | SD rats | MCAO | Curcumin | Intraperitoneal administration. | 50 mg/kg | At 1 h after stroke. | Improve neurologic behavior. | Increase the expression of UCH-L1, ICDH, adenosylhomocysteinase, and eIF4A, and decrease the expression of PLPP. |
| (Gim et al., 2015) | SD rats | MCAO | Curcumin | Intraperitoneal administration. | 50 mg/kg | At 1 h after stroke. | Neuroprotective effect. | Increase the expression of γ-enolase expression. |
| (Li et al., 2016) | Wistar rats | MCAO/R | Curcumin | Intraperitoneal administration. | 300 mg/kg | At 30 min after occlusion. | Reduce brain edema and neurological dysfunction | Down-regulate NF-κB and elevating Nrf2. |
| (Miao et al., 2016) | SD rats | MCAO/R | Curcumin | Intraperitoneal administration. | 50 mg/kg | Once daily for 5 days before MCAO. | Attenuate neurologic dysfunction, infarct volume, and brain water content. | Attenuate inflammation and mitochondrial dysfunction via activation of SIRT1. |
| (Li et al., 2015a) | SD rats | MCAO/R | Curcumin | Intraperitoneal administration. | 80 mg/kg | Once, starting at 0.5 h prior to reperfusion. | Improve neurological score, reduce infarct size, and brain edema. | Activate JAK2/STAT3 signaling pathway. |
| (Shah et al., 2015) | SD rats | MCAO | Curcumin | Intraperitoneal administration. | 50 mg/kg | At 1 h after the onset of MCAO. | Neuroprotective effect. | Increase PP2A subunit B levels. |
| (Wu et al., 2015) | Primary rat cortical neurons | OGD/R | Curcumin | Add to the culture medium. | 5 and 10 μM | Exposed to curcumin (10 μM) for 24 h followed by OGD/R; at the beginning of reoxygenation and maintained for 24 h | Reduce cell injury, improve the anti-oxidative ability of neurons | Activate the expression of thioredoxin in the Nrf2 pathway. |
| (Liu et al., 2014) | SD rats | MCAO/R | Curcumin | Intraperitoneal administration. | 50 and 100 mg/kg | Prior to onset of occlusion for 5 days. | Improve neurological deficit, diminish infarct volume, and increase the number of NeuN-labeled neurons. | Increase mitochondrial biogenesis by elevating NRF-1, mitochondrial UCP2, TFAM and mitochondrial number. |
| (Tu et al., 2014) | SD rats | MCAO | Curcumin | Intraperitoneal administration. | 10 and 50 mg/kg | At 2 and 12 h after the onset of ischemia. | Improve neurological score, neuronal damage, brain water content and reduce infarct size. | Inhibit TLR2/4-NF-κB signaling pathway, reduce MPO activity, and attenuate the release of TNF-α and IL-1β. |
| (Dong et al., 2014) | Brain microvascular endothelial cells | OGD | Curcumin | Add to the culture medium. | 20 μM | Add at 30 min before OGD exposure | Attenuate cell injury, reduce the release of LDH. | Inhibit MAPK pathway and NF-κB activation, reduce IL-1β. |
| (Liu et al., 2013) | SD rats | MCAO/R | Curcumin | Intraperitoneal administration. | 200 mg/kg | For 3 days. The last injection is 2 h prior to surgery. | Improve neurologic behavior, reduce infarct volume. | Upregulate PPAR γ expression and PPAR γ -PPRE binding activity; decrease inflammatory mediators, such as IL-1 β, TNF-α, PGE2, NO, COX-2, and iNOS; suppress IκB degradation. |
| (Wu et al., 2013) | SD rats | MCAO/R | Curcumin | Intraperitoneal administration. | 300 mg/kg | At reperfusion onset. | Reduce infarct volume, cell injury and oxidative stress. | Regulation of PI3K/Akt/Nrf2 signal pathway, reduce the expression of NQO1. |
| (Wang et al., 2013) | Rat brain microvascular endothelial cells | OGD | Curcumin | Add to the culture medium. | 1, 20, and 100 μM | Add prior to OGD. | Attenuate disruption of paracellular permeability. | Protect disruption of TJ and barrier dysfunction via the HO-1 pathway. |
| (Funk et al., 2013) | SD rats | MCAO/R | Curcuminoids | Intraperitoneal administration. | 300 mg/kg | 1 hour prior to reperfusion. | Reduce infarct size, brain edema and neurological function. | Inhibit number and activation state (CD11b and ROS) of neutrophils; decrease the expression of TNFα and ICAM-1, a marker of endothelial activation; reduce NF-κB activation and subsequent ICAM-1 gene expression. |
| (Kakkar et al., 2013) | Wistar rats | BCCAO | Curcumin loaded solid lipid nanoparticles | Oral administration. | 25 and 50 mg/kg | 5 days prior and continued for 3 days after BCCAO | Improve neurological score. | Increase the level of SOD, CAT, GSH, and mitochondrial complex enzyme activities, decrease the level of lipid peroxidation, nitrite, and AChE. |

AchE: acetylcholinesterase; BBB: blood-brain barrier; BCCAO: bilateral common carotid artery occlusion; CAT: catalase; CIRI: cerebral ischemia-reperfusion injury; COX-2: cyclooxygenase-2; dMCAO: distal middle cerebral artery occlusion; GCI: global cerebral ischemia; GPX: glutathione peroxidase; HO-1: heme oxygenase-1; H/R: hypoxia/reoxygenation; ICDH: isocitrate dehydrogenase; IFN-γ: interferon-γ; IL: interleukin; iNOS: inducible nitric oxide synthase; JAK2: Janus kinase 2; LDH: lactate dehydrogenase; LPS: lipopolysaccharide; MCAO/R: middle cerebral artery occlusion and reperfusion; MDA: malondialdehyde; MMP: mitochondrial membrane potential; NF-κB: nuclear factor kappa B; NMDAR1: N-methyl-D-aspartate receptor 1; NO: nitric oxide; NQO1: NAD(P)H: quinone oxidoreductase 1; NRF-1: nuclear respiratory factor-1; Nrf2: nuclear factor-erythroid-related factor-2; NSCs: neural stem cells; OGD/R: oxygen-glucose-deprivation/reperfusion; PGE2: prostaglandin E2; PLPP: pyridoxal phosphate phosphatase; PP2A: protein phosphatase 2A; PPAR γ: peroxisome proliferator-activated receptor γ; ROS: reactive oxygen species; SOD2: type-2 superoxide dismutase; SP1: specific protein 1; STAT3: signal transducer and activator 3 of transcription; TFAM: mitochondrial transcription factor A; TJ: tight junction; TLR: toll-like receptors; TNF: tumor necrosis factor; UCH-L1: ubiquitin carboxy-terminal hydrolase L1; UCP2: uncoupling protein 2; ZO-1: zonula occludens-1.
